# Anomalous dynamics of a passive droplet in active turbulence

**DOI:** 10.1038/s41467-024-47727-1

**Published:** 2024-05-02

**Authors:** Chamkor Singh, Abhishek Chaudhuri

**Affiliations:** 1 Department of Physics, Central University of Punjab, Bathinda, India; 2 Department of Physical Sciences, Indian Institute of Science Education and Research (IISER) Mohali, Sector 81, SAS Nagar, Mohali, Punjab 140306 India

**Keywords:** Biological physics, Computational biophysics

## Abstract

Motion of a passive deformable object in an active environment serves as a representative of both in-vivo systems such as intracellular particle motion in Acanthamoeba castellanii, or in-vitro systems such as suspension of beads inside dense swarms of Escherichia coli. Theoretical modeling of such systems is challenging due to the requirement of well resolved hydrodynamics which can explore the spatiotemporal correlations around the suspended passive object in the active fluid. We address this critical lack of understanding using coupled hydrodynamic equations for nematic liquid crystals with finite active stress to model the active bath, and a suspended nematic droplet with zero activity. The droplet undergoes deformation fluctuations and its movement shows periods of “runs” and “stays”. At relatively low interfacial tension, the droplet begins to break and mix with the outer active bath. We establish that the motion of the droplet is influenced by the interplay of spatial correlations of the flow and the size of the droplet. The mean square displacement shows a transition from ballistic to normal diffusion which depends on the droplet size. We discuss this transition in relation to spatiotemporal scales associated with velocity correlations of the active bath and the droplet.

## Abstract (web summary)

The fluctuating dynamics of a passive object suspended in an active fluid can provide fundamental insight into the fundamental non-equilibrium behavior of the fluid. Singh and Chaudhuri theoretically investigate the dynamics of a passive deformable droplet in active nematic turbulence and show how the motion of the droplet is influenced by the interplay of spatial correlations of the flow and the size of the droplet.

## Introduction

The random motion of a passive tracer particle moving in a fluid at equilibrium is the starting point of describing fluctuating dynamics^1^. Microrheology suggests that the local and bulk mechanical properties of complex fluids can be extracted from the dynamics of such tracer particles^2,3^. Further intricacies arise if the surrounding fluid is a non-equilibrium active bath, like the cell cytosol, which consists of different kinds of semiflexible filaments and motor proteins rendered active by the hydrolysis of chemical fuel like ATP. The dynamics of passive deformable objects in such an active environment can provide invaluable information about the viscoelastic properties of the surrounding fluid and the transport of intracellular vesicles and granules through the cytosol^4^. The active cytosol belongs to the larger class of active fluids, where chemical energy is converted into mechanical work by generating stresses at the microscale^5–8^. Active fluids have been shown to exhibit unusual transport properties such as enhanced diffusion, accumulation near boundaries, and rectification^9,10^. Experiments using passive particles in active fluids suggest anomalous behavior of the passive particle at short time scales, and a dramatically enhanced translational diffusion at long times^11–15^.

In an active fluid, the collective dynamics of the suspended swimmers such as microbial suspensions, cytoskeletal suspensions, self-propelled colloids, and cell tissues, generate spontaneous flows. These flows are characterized by chaotic spatiotemporal patterns and are referred to as active turbulence^16,17^ due to their apparent resemblance with classical turbulence. Experimental studies of active turbulence look at the velocity fields and their statistics to characterize hydrodynamic flows^18^ and show the creation, transportation, and annihilation of topological defects by active stresses^19^. Theoretical studies of passive tracers in active fluids use either the generalized Langevin equation with different properties of the friction and fluctuating force due to the fluid or use particle-based simulations of passive and active objects^20,20–35^, rarely coupling the hydrodynamics of the outer active bath with the suspended passive components because of the complexity involved^27,36^ with notable exceptions^37–41^. Recent experiments of mobile *rigid* inclusions in an active nematic system have further shown the importance of the complex interplay and feedback between the motion of such inclusion and the active fluid^42^. A vesicle moving in the cytosol on the other hand is *deformable* and naturally one would like to understand the influence of the active environment on its dynamics.

**Fig. 1:**
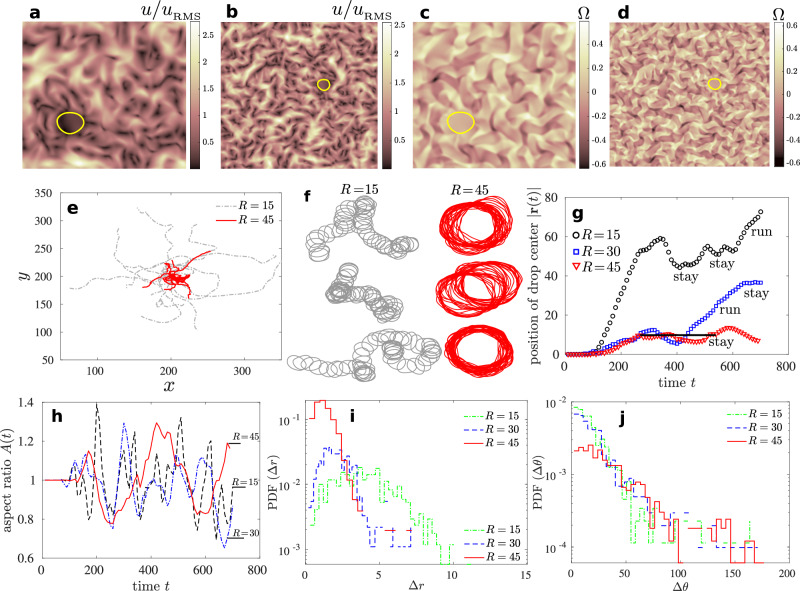
Droplet size dependence and deformation fluctuations. **a**, **b** Typical absolute velocity field scaled by its root mean square value for system size 200 × 200 and 400 × 400, respectively at *t* = 500. The interface between the passive nematic droplet and the outer active bath is shown with a solid yellow line. **c**, **d** Typical vorticity field for system size 200 × 200 and 400 × 400 respectively at *t* = 500. The length and times scales are expressed in units of ${x}_{o}=\sqrt{\frac{{K}_{o}}{{\Gamma }_{o}{C}_{o}{\eta }_{o}}}$ and ${t}_{o}=\frac{1}{{\Gamma }_{o}{C}_{o}}$ respectively, where *K*_*o*_ is the elastic constant, Γ_*o*_ is the molecular relaxation parameter, *C*_*o*_ is a material constant, and *η*_*o*_ is the viscosity in the outer active nematic phase (see Methods). **e** Individual trajectories of the geometric center **r**(*t*) of passive nematic droplet of radius *R* = 15 and *R* = 45, and (**f**) droplet interface during typical realizations for *R* = 15 and *R* = 45. **g** Typical realizations of the position of the droplet center relative to the initial position, ∣**r**(*t*)∣, exhibiting periods of “run" and “stay". **h** Ratio of the horizontal to the vertical span of the droplet for *R* = 15, 30, and 45. The dynamics of translations as well as the aspect ratio slow down as the radius is increased. **i** Probability distribution function of the translational steps taken by the geometric center of the droplet. PDF(Δ*r*) narrows down upon increasing the radius of the droplet. **j** PDF of discrete changes in the direction of motion of the droplet. PDF(Δ*θ*) scales nearly exponentially for intermediate range of angles (Δ*θ* ≈ 25^*o*^ to 65^*o*^) with heavy-tailed rare events for > 90^*o*^ or close to complete reversal. The quantities Δ*r* and Δ*θ* are calculated over a fixed time step of 10^–3^ measured in units of *t*_*o*_.

One of the theoretical approaches to studying active turbulence is active liquid crystal theory, where the continuum equations of motion are built from symmetry arguments. A particular class is active apolar nematic systems characterized by head-tail symmetry of the constituent particles–tending to align with each other. These systems exhibit characteristics such as instability of the isotropic nematic state in certain parameter regimes leading to intriguing pattern formation^6,16,43–45^, universal scalings of the energy spectra^46–48^, topological chaos^49^, and in general far from equilibrium dynamics^50^. The continuum equations for such a system are described in detail in the “Methods” section. The homogeneous isotropic state, in certain parameter regimes, is unstable and goes into a sequence of instabilities^51^. Once the system is in a developed state (Fig. 1a–d), the ensemble-averaged mean kinetic energy in the system approaches a statistically steady state^52^ (Supplementary Fig. 1). We study the dynamics of a passive nematic droplet moving in this statistically stationary state of active nematic turbulence (Fig. 1a–d). Inverse to this setup are systems consisting of active droplets immersed in passive media which demonstrate complex behaviors, for example, self locomotion, spontaneous rotation and division of active nematic droplets^53–55^, self-organization and division in active liquid droplets^56^, dynamic defect structures in active nematic shells^57^, active wetting^58^ and emulsification in mixtures of active and passive component media^59^. As an example, it is known that an isotropic liquid droplet in a passive nematic liquid crystal under applied shear or otherwise undergoes different modes of movement (oscillatory, breakup, or motile) when the activity and anchoring conditions at the surface of the droplet are changed^60,61^.

However, in our system, the suspended phase is a passive nematic soft object with finite interfacial tension, and the bath is an active liquid crystal in a turbulent state. We demonstrate how the interaction between the size of the inclusions and the spatial correlations of the flow affects the mobility of passive inclusions in active turbulence as the surface tension and droplet size are changed. We establish that the velocities in the bath around the droplet are correlated over a certain spatial scale which is independent of the droplet size. However, the velocity autocorrelations of the resulting droplet trajectories exhibit multiple temporal scales which depend on the droplet size, but not so much on the interfacial tension. We discuss the importance of these scales in understanding the dynamics of the droplet in the active bath. Usually, these correlations are neglected for simplicity but we resolve them and posit that these correlations are significant enough to alter the dynamics of the suspended passive droplet. In general, we observe that the droplet undergoes deformation fluctuations and shows periods of “runs" and “stays", much like a bacterium. This aspect, however, strongly depends on the size of the object and the correlations inside the surrounding active bath. We compute an integral length scale associated with the one-time two-point velocity correlation function of the surrounding active bath and observe that the droplets with sizes smaller than this length scale move relatively farther. Finally, we provide a consistent Langevin framework with a random force that is correlated in time. This description describes the simulation results qualitatively well.

## Results

To study the motion of a passive nematic droplet in an active nematic medium, we computationally solve the continuum hydrodynamic and nematodynamic equations for apolar active nematics. The detailed model is described in the Methods section. The essential aspect of our simulations is that the active stresses given as ***σ*** ^A^ = − *ζ***Q** where **Q** characterizes nematic order, are non-zero in the outer bath (*ζ* ≠ 0) or in the active nematic phase surrounding the droplet, and are set to zero inside the droplet (*ζ*  =  0) which is considered as a passive nematic phase. In this work, we restrict ourselves to extensile systems only i.e. the activity parameter is taken to be *ζ*≥0. Initializing from a perturbed nematic state, and upon fine-tuning of the amount of local energy injection (the activity) and other model parameters, the outer phase transitions to a developed active nematic turbulent state (Fig. 1a–d) after some initial period 0 ≤ *t* ≤ 300 of instability growth. Eventually active stresses, on average, are balanced by the passive, dissipative, frictional, and interfacial stresses and the system reaches a statistically steady state (Supplementary Fig. 1).

To characterize the active turbulent state, we first look at the kinetic energy spectrum $E(k)=\langle \frac{1}{2\pi }{\sum }_{k\le | {{{{{{{\bf{k}}}}}}}}| < k+\Delta k}| \hat{{{{{{{{\bf{u}}}}}}}}}({{{{{{{\bf{k}}}}}}}},t){| }^{2}\rangle$ where *k* is the magnitude of the wavevector or the wavenumber, $\hat{{{{{{{{\bf{u}}}}}}}}}({{{{{{{\bf{k}}}}}}}},t)=\int\,{{{{{{{\bf{u}}}}}}}}({{{{{{{\bf{r}}}}}}}},t)\exp (-i{{{{{{{\bf{k}}}}}}}}\cdot {{{{{{{\bf{r}}}}}}}}){{{{{\rm{d}}}}}}{{{{{{{\bf{r}}}}}}}}$, **u** is the velocity field, and 〈〉 denotes average over uncorrelated configurations. We find a power law scaling *E*(*k*) ~ *k*^−4^ (Supplementary Fig. 2) which has been recently observed in a number of active nematic single-phase studies^62,63^. The suspended droplet is also setup to have certain interfacial tension which acts at the interface between the droplet and the outer medium (see Methods for a detailed discussion). We characterize the dynamics of this biologically relevant inverted system by analyzing the trajectories of the droplet with varying radius and interfacial tension, spectrum analysis of the time series of the trajectories, computing the translational and angular step distributions, mean square displacement, time scales associated with velocity autocorrelation function (VACF) of the trajectories, and most importantly the integral length scale associated with the one-time two-point velocity correlation function of the surrounding active bath.

### Statistics of droplet trajectories, time series, and spectral analysis

Droplets of radii in the range 8 ≤ *R* ≤ 64 are suspended in the active turbulent state, and for a given radius the simulations are repeated at least 30 times to obtain an ensemble of stochastic trajectories executed by the droplet. A typical set of trajectories of the geometric center of the droplet is shown in Fig. 1e, f for radius *R* = 15 and *R* = 45. To be precise, the position of the center of the droplet is defined as **r**(*t*) = ∑_*i*_**x**_*i*_(*t*)/*N* where the perimeter of the droplet is divided into *N* points having positions **x**_*i*_ – consistent with the front-tracking algorithm which we use to model the interfacial dynamics^64–66^. As the droplet interacts and is forced by the surrounding active bath, we measure its distance relative to the initial position with time ∣**r**(*t*)∣; typical cases are depicted in Fig. 1g. Along the trajectories, the droplets undergo periods of “runs" and “stays" as marked on the plot.

**Fig. 2:**
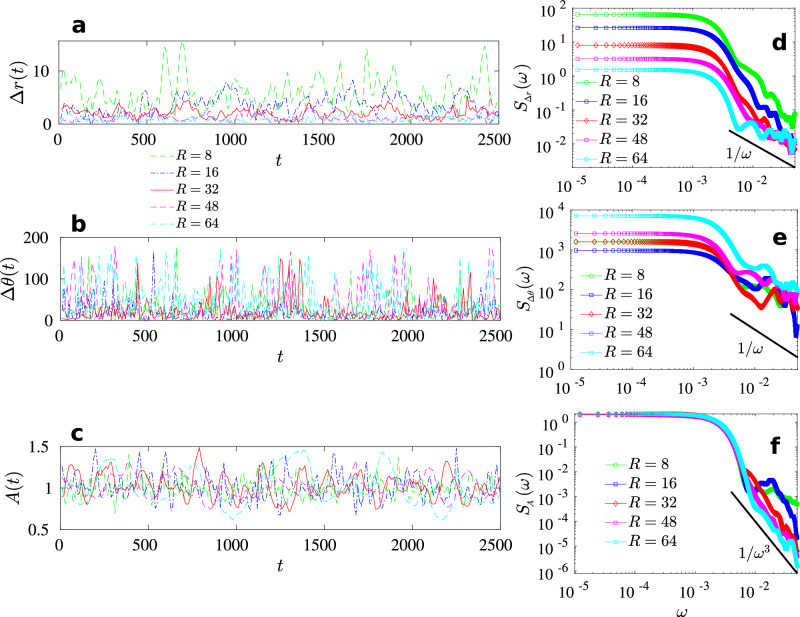
Spectral analysis of droplet variables. The time series of (**a**) translational step size Δ*r*, (**b**) change in direction of motion Δ*θ*, and (**c**) droplet aspect ratio or deformation parameter *A*, respectively. **d**–**f** Corresponding power spectrum of the time series shown in (**a**–**c**). The spectrum in panel (**d**) implies that two consecutive droplet position increments are not independent and thus the case is different from standard Brownian increments because Brownian increments are known to be independent. Similarly, panel (**e**) implies that two consecutive Δ*θ* are not independent. The spectrum of the time series of aspect ratio *A* indicates that the droplet deformations have underlying oscillatory features. The solid black lines in (**d**–**f**) are a guide to the eye.

We also note that the period of undulations of droplet deformation parameter *A*(*t*), defined as the ratio of horizontal to vertical span of the droplet, increases as we increase the radius (Figs. 1h and  2c). With increasing size, the droplet dynamics slows down and we transit from a “fast" process towards a “slow" process.

**Fig. 3:**
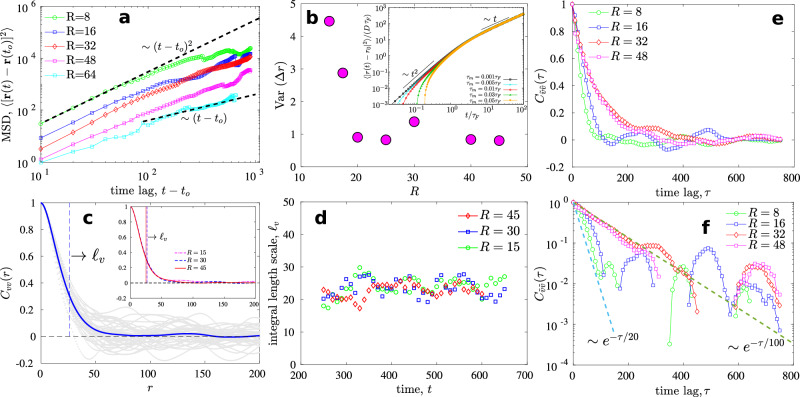
Dynamics and spatiotemporal scales. **a** Mean square displacement (MSD) of droplets of different radii *R* with *t*_*o*_ = 300. **b** Variance of discrete step sizes taken by droplets of different radii. The variance decreases as the droplet radius increases. (**b**, inset) An analytical calculation of the MSD shows crossover from ballistic to diffusive regime for a particle under exponentially correlated random force. MSD is shown for different values of the mass *m*, which is varied by choosing five different inertial times *τ*_*m*_( = *m*/*α*), in the limit *τ*_*m*_ ≪ *τ*_*F*_ (see Methods for details). The crossover depends on *τ*_*F*_ and also on the droplet size *R*. **c** Time averaged one-time two-point velocity correlation function *C*_*vv*_(*r*) of the velocity field in the active nematic bath. The integral length scale ${\ell }_{v}\equiv \int\nolimits_{0}^{\infty }{C}_{vv}(r){{{{{\rm{d}}}}}}r$ is marked with a vertical dashed line; (**c**, inset) *ℓ*_*v*_ in the active bath changes negligibly upon changing the droplet radius. **d** Integral length scale with time during typical realizations. **e** VACF ${C}_{\tilde{v}\tilde{v}}(\tau )$ of the droplet trajectories with varying *R*, and (**f**) the same on a Semi-logarithmic scale resolving the presence of multiple time scales.

The trajectories consist of discrete translational steps taken by the droplet center Δ*r* ≡ ∣**r**(*t* + Δ*t*) − **r**(*t*)∣ in simulation time step Δ*t*. In addition to translational steps Δ*r*, we define angular steps $\Delta \theta={\cos }^{-1}\left[{{{{{{{\bf{a}}}}}}}}\cdot {{{{{{{\bf{b}}}}}}}}/ab\right]$ where **a** = **r**(*t* + Δ*t*) − **r**(*t*) and **b** = **r**(*t*) − **r**(*t* − Δ*t*). The distribution of these two quantities is shown in Fig. 1i, j, and their time series in shown in Fig. 2a, b. The step size distribution (or equivalently the step speed distribution) spreads upon decreasing the radius of the droplet (Fig. 1i). Smaller droplets execute relatively larger translational steps (Figs. 1i, 2a) which is clear from the increasing variance of Δ*r* as we decrease *R* (Fig. 3b). The change in direction of motion of the droplet is nearly exponentially distributed for intermediate range of angles (Δ*θ* ≈ 25^*o*^ to 65^*o*^), and exhibits some rare events of > 90^*o*^ or close to complete reversal of the direction (Figs. 1j and 2b).

Figure 2(a–c) depicts the time series of (a) translational step size Δ*r*(*t*), (b) change in the direction of motion Δ*θ*(*t*), and (c) droplet aspect ratio *A*(*t*) respectively, while Fig. 2 (d–f) shows the corresponding power spectrum of these signals. Smaller droplets “drift" significantly more than the larger ones. The deformation time scales also change (Fig. 2c). The corresponding spectral plots indicate that the translational step and the change in the direction of motion exhibit nearly 1/*ω* or pink noise at higher frequencies. The spectrum of the aspect ratio or the deformation parameter *A*(*t*), however, exhibits nearly 1/*ω*^3^ noise. Although it is known that 1/*ω* noise is present in many natural, man-made, and scientific systems^67,68^, its underpinnings are debated over decades^69,70^. Does the above 1/*ω* noise have to do with self-organized states in our system, or do they have a different origin? In addition, 1/*ω*^3^ noise in the deformation or the droplet aspect ratio spectrum indicates that deformation dynamics are distinct from the motion of the droplet. This type of noise is usually observed in oscillators implying that the droplet deformation has underlying oscillatory features. Figure 2d, e implies that neither two consecutive droplet position increments nor two consecutive Δ*θ* are independent, implying that the dynamics are far from Brownian.

### Mean square displacements and spatiotemporal correlations

Assuming a functional form for the translational distribution $f(\Delta r)={{{{{{{\mathcal{B}}}}}}}}{e}^{-\lambda {(\Delta r)}^{2}}$ with ${{{{{{{\mathcal{B}}}}}}}}$ a constant, the translational diffusion coefficient comes out to be $D=\int\nolimits_{-\infty }^{\infty }{(\Delta r)}^{2}f(\Delta r)d(\Delta r)/2\tau=\sqrt{\pi }{{{{{{{\mathcal{B}}}}}}}}/(4\tau {\lambda }^{3/2})$ with discretization time *τ* and a diffusive mean square displacement (MSD) ~ *Dt* for *t* ≫ *τ*. However, our computation of MSD shows a regime where 〈Δ**r**^2^(*t*)〉 ~ *t*^*δ*^ with power exponent 1 < *δ* < 2, before any transition to normal diffusion (Fig. 3a). This behavior has been observed in a number of experiments of tracer diffusion in bacterial turbulence^11,71–74^. Also, the transition time depends on *R,* as depicted in Fig. 3a.

To understand how the droplet of a given size interacts with the active bath, we compute one-time two-point velocity correlation function ${C}_{vv}(r)=\langle {u}_{i}(0){u}_{i}(r)\rangle /\langle {({u}_{i}(0))}^{2}\rangle$ of the velocity field of the active bath, and associated integral length scale, ${\ell }_{v}\equiv \int\nolimits_{0}^{\infty }{C}_{vv}(r){{{{{\rm{d}}}}}}r$ (Fig. 3c, d). We find that as we decrease the radius of the droplet below *ℓ*_*v*_, the droplet executes relatively larger Δ*r* (Figs. 3b, 2a, and  1i). Conversely, droplets with a size larger than *ℓ*_*v*_ undergo negligible overall displacement. The variance of step size distribution drops as the size of the droplet is increased, and if *R* > *ℓ*_*v*_ the variance drops to ≈ 1 (Fig. 3b). We propose that droplets with *R* ≪ *ℓ*_*v*_ experience relatively larger drifts and droplets with *R* ≫ *ℓ*_*v*_ move relatively smaller distances because in the latter case the velocities at any two opposite ends of the droplet are expected to be decorrelated – resulting in negligible net advection of the droplet.

To further understand the droplet dynamics, we compute the normalized VACF ${C}_{\tilde{v}\tilde{v}}(\tau )=\langle {\tilde{v}}_{i}(t){\tilde{v}}_{i}(t+\tau )\rangle /\langle {({\tilde{v}}_{i}(t))}^{2}\rangle$, where ${\tilde{v}}_{i}$ is the *i*^th^ component of the droplet velocity (Fig. 3e, f). As the droplet radius increases, ${C}_{\tilde{v}\tilde{v}}(\tau )$ decays slower with lag time *τ* indicating a dependance on the size of the droplet, as we note in Fig. 3 (e, f). We also note the existence of multiple time scales (Fig. 3f), which again distinguish the present droplet dynamics from standard Brownian VACF. It is also to be noted that although the change in radius alters the time scale associated with the VACF (Fig. 3e, f), it has negligible influence on *ℓ*_*v*_ (Fig. 3c, inset) implying that suspending a droplet has negligible effect on the velocity characteristics of the surrounding active bath.

### Effect of interfacial tension

**Fig. 4:**
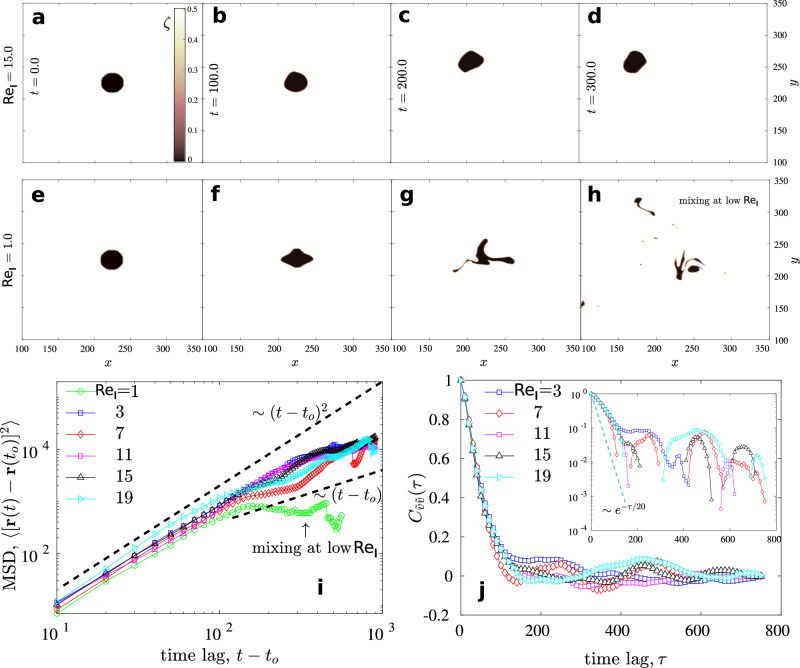
Effect of interfacial tension. The droplet is viewed at different time points using the activity field *ζ* for (**a**–**d**) ${{{{{{{{\rm{Re}}}}}}}}}_{{{{{{{{\rm{I}}}}}}}}}=15$ and (**e**–**h**) ${{{{{{{{\rm{Re}}}}}}}}}_{{{{{{{{\rm{I}}}}}}}}}=1$. At the lower value of ${{{{{{{{\rm{Re}}}}}}}}}_{{{{{{{{\rm{I}}}}}}}}}$, we show a break up of the droplet and subsequent mixing in the outer active bath. The color bar shows the strength of the activity field *ζ*. **i** MSD with varying droplet interfacial tension ${{{{{{{{\rm{Re}}}}}}}}}_{{{{{{{{\rm{I}}}}}}}}}$ showing the initial ballistic regime and the transition towards a diffusive regime. The dashed lines are a guide to the eye. At very low ${{{{{{{{\rm{Re}}}}}}}}}_{{{{{{{{\rm{I}}}}}}}}}=1$, MSD reaches a plateau, indicating that the center of mass of the mixed sub-droplets remains almost stationary after the initial ballistic regime. **j** The VACF with varying ${{{{{{{{\rm{Re}}}}}}}}}_{{{{{{{{\rm{I}}}}}}}}}$ indicates that varying the surface tension does not give rise to different time scales in the VACF for a given droplet size. In all the plots, *R* = 16.

We compute the MSD and the VACF with varying ratios of the interfacial tension to the viscous force given as ${{{{{{{{\rm{Re}}}}}}}}}_{{{{{{{{\rm{I}}}}}}}}}$ (see Methods). In Figs. 4a–h, we show the droplet shape for two different ${{{{{{{{\rm{Re}}}}}}}}}_{{{{{{{{\rm{I}}}}}}}}}$ as time progresses. For relatively lower surface tension, the droplet begins to break and mix with the outer active bath (Fig. 4e–h). The MSD exhibits a prolonged ballistic regime and transitions to a regime with MSD ~ *t*^*δ*^ with 1 < *δ* < 2 (Fig. 4i). The exception occurs for very small values of ${{{{{{{{\rm{Re}}}}}}}}}_{{{{{{{{\rm{I}}}}}}}}}$. The MSD for this mixing case does not become diffusive and reaches to a plateau, indicating that the center of mass of the mixed sub-droplets remains almost stationary after the initial ballistic regime. Contrary to the size effect in Fig. 3, varying ${{{{{{{{\rm{Re}}}}}}}}}_{{{{{{{{\rm{I}}}}}}}}}$ does not give rise to appreciably different time scales for VACF (Fig. 4j). The effect of ${{{{{{{{\rm{Re}}}}}}}}}_{{{{{{{{\rm{I}}}}}}}}}$ and *R* on droplet trajectories is also explored with the help of ensemble-averaged total length *l* of trajectory divided by the time period of the trajectory *T*  i.e.  〈*l*/*T*〉 (Supplementary Figs. 3 and  4). Although 〈*l*/*T*〉 comes out to be inversely proportional to radius *R*, which endorses our postulate that the smaller droplets travel longer in a given time period, ${{{{{{{{\rm{Re}}}}}}}}}_{{{{{{{{\rm{I}}}}}}}}}$ has a negligible effect on 〈*l*/*T*〉, except in cases where droplet breaks and mixes.

### Effective Langevin dynamics

Our observations suggest that the random forces due to the active bath on the droplet are correlated and depend on the droplet size. Therefore, we assume the following form of a random force acting on the droplet with a memory which decays exponentially in time,

1 $$\langle F({t}^{{\prime} })F({t}^{{\prime\prime} })\rangle=\Lambda \exp [-| {t}^{{\prime} }-{t}^{{\prime\prime} }| /{\tau }_{F}],\,\langle F(t)\rangle=0,$$

where *τ*_*F*_ is a characteristic time scale, Λ ≡ *Dα*^2^/*τ*_*F*_, where *D* is the diffusion coefficient and *α* is the coefficient of friction from the Langevin equation for the athermal case $m\ddot{r}(t)=-\alpha \dot{r}(t)+F(t)$ (see Methods for details with the thermal noise). The MSD 〈*r*(*t*)^2^〉 then can be obtained by integrating the Langevin equation with exponentially correlated random force and is depicted in (Fig. 3b, inset). The crossover from a ballistic 〈*r*(*t*)^2^〉 ~ *t*^2^ to diffusive 〈*r*(*t*)^2^〉 ~ *t* is observed. In the limit *τ*_*F*_ ≫ *τ*_*m*_, the steady-state velocity autocorrelation is given as

2 $$\langle {{{{{{{\bf{v}}}}}}}}(t)\cdot {{{{{{{\bf{v}}}}}}}}(0)\rangle=\frac{2D}{{\tau }_{F}}\exp [-t/{\tau }_{F}].$$

where *τ*_*m*_ ≡ *m*/*α* is the inertial time scale. A comparison of this with the VACF obtained from our simulations suggests that *τ*_*F*_ should increase with *R* shown in simulations in Fig. 3e, f. In the previous section, 〈*l*/*T*〉 ~ 1/*R* suggests that the average velocity of the droplet decreases approximately linearly with the droplet size (Supplementary Fig. 3). From the effective Langevin approach, we note that the mean squared velocity in the long time limit for the athermal case can be obtained as $\langle {v}^{2}\rangle={u}_{0}^{2}/\left[1+m/\alpha {\tau }_{F}\right]$, where ${u}_{0}=\sqrt{2D/{\tau }_{F}}$ is a characteristic active velocity of the particle (see Methods for details). Therefore, we can conclude that larger particles will have smaller absolute velocities.

Effective Langevin descriptions of tracers in active baths have been used in the past^11,75–79^. For instance, Wu and Libchaber^11^ used similar approach to relate the tracer dynamics with standard Brownian motion but concluded that the presence of collective effects in the outer bath are also important. It is important to note that the effective Langevin description can only qualitatively reproduce some aspects of the droplet motion. Our numerical simulations have shown *how* collective effects in the bath affect the dynamics of inclusions with the help of computations of spatial correlations of the velocity field of the active bath relative to inclusion size, VACF, MSD, and spectral analysis. These results need to be integrated into a more detailed theoretical study.

## Discussion

The study of active fluids and their interaction with inclusions can provide us with possibilities to harness the inherently chaotic dynamics of active fluids to perform useful functions^42^. Motile bacteria is known to interact with inclusions generating asymmetric forces, while rigid inclusions in active nematic fluid made using biofilaments such as microtubules, can rotate milli-meter sized propellers and pinwheels. However, intracellular objects in biologically active fluids are deformable in nature. To this end in this work, we resolved to understand the interaction between active fluids and motile and deformable inclusions.

We have studied the stochastic dynamics of a passive nematic droplet suspended in a statistically steady state of active nematic turbulence. The transition from superdiffusive mean square displacement regime to normal diffusion depends on the size of the suspension and the properties of the active bath. We find that the integral length scale associated with the two-point velocity correlation function in an active biological medium can be used to determine whether a passive object with a particular size will remain almost positionally localized or will experience considerable “drift". The motion comes out to be far from the equilibrium standard Brownian motion. Few aspects of the dynamics can be qualitatively described by a Langevin model with exponentially correlated random force, however, for full understanding one needs to resolve the spatiotemporal correlations in the surrounding active bath.

Our results can be experimentally tested, for example, using a setup of quasi-two-dimensional suspension of beads and bacteria on a soap film^11^ or inside thin fluid films^71^. Experiments on self-diffusion of the tagged bacterium in dense swarms grown on agar plates^72^, or of ameboid cells^73^, already confirm the existence of a superdiffusive regime where it is hard to reach a normal diffusive regime. For example, multi-potent progenitor cells exhibit superdiffusive regime which can be persistent even for hours^74^. Similar observations have been made in setup of quasi-two-dimensional suspension of beads and bacteria on a soap film^11^ or inside thin fluid films^71^. These experimental observations of superdiffusion and delayed transition to normal diffusion of suspensions inside active swarms lack a concrete theoretical explanation. Here we have pinpointed how the size of the droplet and the properties of the active bath change the spatial and temporal scales. Our numerical study will establish a better understanding of proposed analytical theories of diffusion of bodies in active systems^12,20,30–35^.

## Methods

### Active nematohydrodynamics simulations

Apolar active systems in the continuum limit can be modeled using the passive nematic liquid crystal theory with added active stresses, combined with momentum hydrodynamics. The orientation of nematics is described by a director field **n**, with **n** ≡ − **n** for apolar nematics. The symmetric traceless second rank tensor ${{{{{{{\bf{Q}}}}}}}}=q\frac{d}{d-1}({{{{{{{\bf{n}}}}}}}}{{{{{{{\bf{n}}}}}}}}-{{{{{{{\bf{I}}}}}}}}/d)$ characterizes the nematic order, where *q* is the strength of the nematic order, **I** is the identity tensor and *d* denotes the spatial dimension. In our simulations, *d* = 2. The evolution of **Q** is described by the nematodynamic equation^43,45^

3 $${\partial }_{t}{{{{{{{\bf{Q}}}}}}}}+{{{{{{{\bf{u}}}}}}}}\cdot \nabla {{{{{{{\bf{Q}}}}}}}}={{{{{{{\bf{S}}}}}}}}+\Gamma {{{{{{{\bf{H}}}}}}}}$$

where **S** = (*λ***E** + **Ω**) ⋅ (**Q** + **I**/*d*) + (**Q** + **I**/*d*) ⋅ (*λ***E** − **Ω**) − 2*λ*(**Q** + **I**/*d*)(**Q** : ∇ **u**) is co-moving tensor with **E** = [ ∇ **u** + (∇**u**)^*T*^]/2 and **Ω** = [ ∇ **u** − (∇**u**)^*T*^]/2 being the strain rate and vorticity tensors respectively. The tensor **H** describes the relaxation of **Q** towards the minimum of a free energy and can be written as **H** = *K*∇^2^**Q** + *C***Q**/3 + *C*(**QQ** − **Q **: **Q** **I**/*d*) − *C***Q**(**Q **: **Q**), where *K* is the elastic constant and *C* is a material constant^43^. The **Q**-tensor field is coupled to the velocity field **u** via the momentum equation

4 $$\rho ({\partial }_{t}{{{{{{{\bf{u}}}}}}}}+{{{{{{{\bf{u}}}}}}}}\cdot \nabla {{{{{{{\bf{u}}}}}}}})=\nabla \cdot (-p{{{{{{{\bf{I}}}}}}}}+2\eta {{{{{{{\bf{E}}}}}}}}+{{{{{{{{\boldsymbol{\sigma }}}}}}}}}^{{{{{{{{\rm{P}}}}}}}}}+{{{{{{{{\boldsymbol{\sigma }}}}}}}}}^{{{{{{{{\rm{A}}}}}}}}})-\mu {{{{{{{\bf{u}}}}}}}}+{{{{{{{{\bf{f}}}}}}}}}^{{{{{{{{\rm{I}}}}}}}}},$$

where the passive elastic stress tensor ***σ***^P^ = 2*λ*(**Q** + **I**/*d*)(**Q** : **H**) − *λ***H** ⋅ (**Q** + **I**/*d*) − *λ*(**Q** + **I**/*d*) ⋅ **H** + **Q** ⋅ **H** − **H** ⋅ **Q** − *K*∂_*i*_*Q*_*kl*_∂_*j*_*Q*_*kl*_^43^. The stress tensor ***σ*** ^A^ = − *ζ***Q** gives rise to an activity or motility in the system in the regions where *ζ* ≠ 0 (outside the droplet) and remains zero in the region occupied by the droplet. We restrict ourselves to extensile systems only i.e. the activity parameter is taken to be *ζ* ≥ 0. The term − *μ***u** mimics substrate friction or drag, and *p* is the pressure. The fluid is considered incompressible so that ∇ ⋅ **u** = 0. The stability of the system of equations around the state **Q** = **0** and **u** = **0** depends upon the activity and alignment parameter strengths, the size of the system, as well as other model parameters. We select the parameters such that the system enters a turbulent state as time progresses. The trajectories of the passive nematic droplet are then studied in this statistically stationary active turbulent state. **f** ^I^ in Eq. (4) is the interfacial force term which we discuss in detail in a later subsection.

### Rescaling of governing equations and simulation parameters

If we consider the following reduced or rescaled variables/fields/properties/operators:

${\rho }^{{\prime} }=\rho /{\rho }_{o},\ {t}^{{\prime} }=t/{t}_{o},\ {\partial }_{t}^{{\prime} }={t}_{o}{\partial }_{t},\ {{{{{{{{\bf{u}}}}}}}}}^{{\prime} }={{{{{{{\bf{u}}}}}}}}/{u}_{o},\ {\nabla }^{{\prime} }={x}_{o}\nabla,\ {p}^{{\prime} }=p/{p}_{o}, \ {\eta }^{{\prime} }= \eta /{\eta }_{o},\ {{{{{{{{\bf{E}}}}}}}}}^{{\prime} }={{{{{{{\bf{E}}}}}}}}/{E}_{o},\ {{{{{{{{{\boldsymbol{\sigma }}}}}}}}}^{{{{{{{{\rm{P}}}}}}}}}}^{{\prime} }={{{{{{{{\boldsymbol{\sigma }}}}}}}}}^{{{{{{{{\rm{P}}}}}}}}}/{\sigma }_{o}^{{{{{{{{\rm{P}}}}}}}}},\ {\zeta }^{{\prime} }=\zeta /{\zeta }_{o},\ {{{{{{{{\bf{Q}}}}}}}}}^{{\prime} }={{{{{{{\bf{Q}}}}}}}}/q,\ {\mu }^{{\prime} }=\mu /{\mu }_{o},\ {{{{{{{{{\bf{f}}}}}}}}}^{{{{{{{{\rm{I}}}}}}}}}}^{{\prime} }={{{{{{{{\bf{f}}}}}}}}}^{{{{{{{{\rm{I}}}}}}}}}/{f}_{o}^{{{{{{{{\rm{I}}}}}}}}},\ {\Gamma }^{{\prime} }= \Gamma /{\Gamma }_{o}, \ {{{{{{{{\bf{H}}}}}}}}}^{{\prime} }={{{{{{{\bf{H}}}}}}}}/{H}_{o},\ {{{{{{{{\bf{S}}}}}}}}}^{{\prime} }= {{{{{{{\bf{S}}}}}}}}/{S}_{o},\ {K}^{{\prime} }=K/{K}_{o},\ {C}^{{\prime} }=C/{C}_{o},$ and fix the following scales: ${x}_{o}=\sqrt{\frac{{K}_{o}}{{\Gamma }_{o}{C}_{o}{\eta }_{o}}},\quad {t}_{o}=\frac{1}{{\Gamma }_{o}{C}_{o}},\quad {\rho }_{o}=\frac{{\eta }_{o}{t}_{o}}{{x}_{o}^{2}},\quad {p}_{o}=\frac{{\eta }_{o}}{{t}_{o}},\quad {u}_{o}= \frac{{x}_{o}}{{t}_{o}},\quad {S}_{o}= \frac{{u}_{o}}{{x}_{o}},\quad {H}_{o}={C}_{o}={\sigma }_{o}^{{{{{{{{\rm{P}}}}}}}}}=\frac{{K}_{o}}{{x}_{o}^{2}},\quad q=1,$ then the dimensional form of the governing equations can be rescaled into the following non-dimensional form

5 $${{{{{{{\rm{Re}}}}}}}}\ {\rho }^{{\prime} }({\partial }_{t}^{{\prime} }{{{{{{{{\bf{u}}}}}}}}}^{{\prime} }+{{{{{{{{\bf{u}}}}}}}}}^{{\prime} }\cdot {\nabla }^{{\prime} }{{{{{{{{\bf{u}}}}}}}}}^{{\prime} })={\nabla }^{{\prime} }\cdot (-{p}^{{\prime} }{{{{{{{\bf{I}}}}}}}}+2{\eta }^{{\prime} }{{{{{{{{\bf{E}}}}}}}}}^{{\prime} }+{{{{{{{{{\boldsymbol{\sigma }}}}}}}}}^{{{{{{{{\rm{P}}}}}}}}}}^{{\prime} }\\ -{{{{{{{{\rm{Re}}}}}}}}}_{{{{{{{{\rm{a}}}}}}}}}\ {\zeta }^{{\prime} }{{{{{{{{\bf{Q}}}}}}}}}^{{\prime} })-{{{{{{{{\rm{Re}}}}}}}}}_{{{{{{{{\rm{f}}}}}}}}}\ {\mu }^{{\prime} }{{{{{{{{\bf{u}}}}}}}}}^{{\prime} }+{{{{{{{{\rm{Re}}}}}}}}}_{{{{{{{{\rm{I}}}}}}}}}\ {{{{{{{{{\bf{f}}}}}}}}}^{{{{{{{{\rm{I}}}}}}}}}}^{{\prime} },$$

6 $${\partial }_{t}^{{\prime} }{{{{{{{{\bf{Q}}}}}}}}}^{{\prime} }+{{{{{{{{\bf{u}}}}}}}}}^{{\prime} }\cdot {\nabla }^{{\prime} }{{{{{{{{\bf{Q}}}}}}}}}^{{\prime} }={{{{{{{{\bf{S}}}}}}}}}^{{\prime} }+{\Gamma }^{{\prime} }{{{{{{{{\bf{H}}}}}}}}}^{{\prime} }$$

7 $${{{{{{{{\bf{E}}}}}}}}}^{{\prime} }=[{\nabla }^{{\prime} }{{{{{{{{\bf{u}}}}}}}}}^{{\prime} }+{({\nabla }^{{\prime} }{{{{{{{{\bf{u}}}}}}}}}^{{\prime} })}^{T}]/2$$

8 $${{{{{{{{\mathbf{\Omega }}}}}}}}}^{{\prime} }=[{\nabla }^{{\prime} }{{{{{{{{\bf{u}}}}}}}}}^{{\prime} }-{({\nabla }^{{\prime} }{{{{{{{{\bf{u}}}}}}}}}^{{\prime} })}^{T}]/2$$

9 $${{{{{{{{\bf{S}}}}}}}}}^{{\prime} }=	 (\lambda {{{{{{{{\bf{E}}}}}}}}}^{{\prime} }+{{{{{{{{\mathbf{\Omega }}}}}}}}}^{{\prime} })\cdot ({{{{{{{{\bf{Q}}}}}}}}}^{{\prime} }+{{{{{{{\bf{I}}}}}}}}/d)+({{{{{{{{\bf{Q}}}}}}}}}^{{\prime} }+{{{{{{{\bf{I}}}}}}}}/d)\cdot (\lambda {{{{{{{{\bf{E}}}}}}}}}^{{\prime} }-{{{{{{{{\mathbf{\Omega }}}}}}}}}^{{\prime} })\\ 	 -2\lambda ({{{{{{{{\bf{Q}}}}}}}}}^{{\prime} }+{{{{{{{\bf{I}}}}}}}}/d)({{{{{{{{\bf{Q}}}}}}}}}^{{\prime} }:{\nabla }^{{\prime} }{{{{{{{{\bf{u}}}}}}}}}^{{\prime} })$$

10 $${{{{{{{{\bf{H}}}}}}}}}^{{\prime} }=	 {K}^{{\prime} }{\nabla }^{{\prime} 2}{{{{{{{{\bf{Q}}}}}}}}}^{{\prime} }+{C}^{{\prime} }{{{{{{{{\bf{Q}}}}}}}}}^{{\prime} }/3+{C}^{{\prime} }({{{{{{{{\bf{Q}}}}}}}}}^{{\prime} }{{{{{{{{\bf{Q}}}}}}}}}^{{\prime} }-{{{{{{{{\bf{Q}}}}}}}}}^{{\prime} }:{{{{{{{{\bf{Q}}}}}}}}}^{{\prime} }\ {{{{{{{\bf{I}}}}}}}}/d)\\ 	 -{C}^{{\prime} }{{{{{{{{\bf{Q}}}}}}}}}^{{\prime} }({{{{{{{{\bf{Q}}}}}}}}}^{{\prime} }:{{{{{{{{\bf{Q}}}}}}}}}^{{\prime} })$$

11 $${{{{{{{{{\boldsymbol{\sigma }}}}}}}}}^{{{{{{{{\rm{P}}}}}}}}}}^{{\prime} }=	 2\lambda ({{{{{{{{\bf{Q}}}}}}}}}^{{\prime} }+{{{{{{{\bf{I}}}}}}}}/d)({{{{{{{{\bf{Q}}}}}}}}}^{{\prime} }:{{{{{{{{\bf{H}}}}}}}}}^{{\prime} })-\lambda {{{{{{{{\bf{H}}}}}}}}}^{{\prime} }\cdot ({{{{{{{{\bf{Q}}}}}}}}}^{{\prime} }+{{{{{{{\bf{I}}}}}}}}/d)\\ 	 -\lambda ({{{{{{{{\bf{Q}}}}}}}}}^{{\prime} }+{{{{{{{\bf{I}}}}}}}}/d)\cdot {{{{{{{{\bf{H}}}}}}}}}^{{\prime} }+{{{{{{{{\bf{Q}}}}}}}}}^{{\prime} }\cdot {{{{{{{{\bf{H}}}}}}}}}^{{\prime} }-{{{{{{{{\bf{H}}}}}}}}}^{{\prime} }\cdot {{{{{{{{\bf{Q}}}}}}}}}^{{\prime} }-{K}^{{\prime} }{\partial }_{i}^{{\prime} }{Q}_{kl}^{{\prime} }{\partial }_{j}^{{\prime} }{Q}_{kl}^{{\prime} }.$$

Here ^′^ denotes a non-dimensional variable/field/property/operator, and

12 $${{{{{{{\rm{Re}}}}}}}}=\frac{{\rho }_{o}{u}_{o}{x}_{o}}{{\eta }_{o}},\quad {{{{{{{{\rm{Re}}}}}}}}}_{{{{{{{{\rm{a}}}}}}}}}=\frac{{t}_{o}{\zeta }_{o}}{{\eta }_{o}},\quad {{{{{{{{\rm{Re}}}}}}}}}_{{{{{{{{\rm{f}}}}}}}}}=\frac{{\mu }_{o}{x}_{o}^{2}}{{\eta }_{o}},\quad {{{{{{{{\rm{Re}}}}}}}}}_{{{{{{{{\rm{I}}}}}}}}}=\frac{{x}_{o}{t}_{o}{f}_{o}^{{{{{{{{\rm{I}}}}}}}}}}{{\eta }_{o}},$$

are non-dimensional groups signifying the ratio of inertial force to viscous force, active force to viscous force, friction force to viscous force, and interfacial tension force to viscous force, respectively. Numerical values of the non-dimensional parameters and their properties in the active and passive phases are summarized in Table 1. In addition to fluid properties in the active and passive phases, the table also summarizes other simulation parameters namely the system size, grid size, time step, and droplet radius.

**Table 1:** Numerical values of parameters and fluid properties adopted in the simulations

Non-dimensional parameter/property		
${{{{{\rm{Re}}}}}}$	0.1	
${{{{{{\rm{Re}}}}}}}_{{{{{{\rm{a}}}}}}}$	0.5	
${{{{{{\rm{Re}}}}}}}_{{{{{{\rm{f}}}}}}}$	0.00075	
${{{{{{\rm{Re}}}}}}}_{{{{{{\rm{I}}}}}}}\equiv {{{{{{\rm{Ca}}}}}}}^{-1}$	1, 3, 7, 11, 15, 19	
System size *L*_*x*_ × *L*_*y*_	200 × 200, 400 × 400, 450 × 450	
Grid size *M* × *N*	256 × 256, 512 × 512, 576 × 576	
Droplet radius *R*	8, 15, 16, 30, 32, 45, 48, 64	
Time step Δ*t*	1.0 × 10^−3^	

	Phase 1: active nematic fluid	Phase 2: passive nematic droplet
Density ${\rho }^{{\prime} }$	${\rho }_{1}^{{\prime} }=1.0$	${\rho }_{2}^{{\prime} }=2.0$
Viscosity ${\eta }^{{\prime} }$	${\eta }_{1}^{{\prime} }=1.0$	${\eta }_{2}^{{\prime} }=2.0$
Activity ${\zeta }^{{\prime} }$	${\zeta }_{1}^{{\prime} }=1.0$	${\zeta }_{2}^{{\prime} }=0.0$
Friction ${\mu }^{{\prime} }$	${\mu }_{1}^{{\prime} }=1.0$	${\mu }_{2}^{{\prime} }=1.0$
Alignment *λ*	*λ*_1_ = 0.8	*λ*_2_ = 0.8
Elastic constant ${K}^{{\prime} }$	${K}_{1}^{{\prime} }=0.25$	${K}_{2}^{{\prime} }=0.25$
Material constant ${C}^{{\prime} }$	${C}_{1}^{{\prime} }=0.4$	${C}_{2}^{{\prime} }=0.4$
Relaxation ${\Gamma }^{{\prime} }$	${\Gamma }_{1}^{{\prime} }=1.0$	${\Gamma }_{2}^{{\prime} }=1.0$

The above set of parameters are close to, for instance, the studies carried out by^43,52^.

The grid and time steps are taken such that the advective, as well as viscous time step conditions, are well satisfied, namely^64^

13 $$\Delta t \, < \, {C}_{1}\frac{\Delta }{{u}_{\max }},\,\,\, \Delta t \, < \, {C}_{2}\frac{2{\eta }_{\min }}{{\rho }_{\max }{u}_{\max }^{2}},\,\,\, \Delta t \, < \, {C}_{3}\frac{{\rho }_{\min }{\Delta }^{2}}{4{\eta }_{\max }},$$

where Δ is the grid size *L*_*x*_/*M* = *L*_*y*_/*N*, and subscripts ${{{{{{{\rm{max,\; min}}}}}}}}$ denote the maximum and minimum values of the respective properties from the two phases. For simulation to remain stable, the factors *C*_1_, *C*_2_, and *C*_3_ have to be < 1, and to be conservative, we choose time and grid size combination such that these conditions are always satisfied.

### Numerical scheme

The coupled set of nematodynamic, momentum, and incompressibility equations are integrated computationally, utilizing a finite-volume based pressure projection algorithm under an Eulerian one-fluid approach^64,66,80^. In this, although the form of the governing equations is same for both the phases, the properties and parameters such as density, viscosity, activity, alignment, friction, etc. are considered as fields and take different values in the two phases i.e. the values of ${\zeta}^{{\prime}}, \, {\eta}^{{\prime}}, \, {\rho}^{{\prime}}, \, {\lambda}^{{\prime}},\, {\mu}^{{\prime}}$ etc. at any given instant of time are set by the location of the interface.

In addition, the interface exerts a stress of magnitude *γκ* in a direction **n** normal to the interface where *γ* is the (constant) interfacial tension and *κ* is the magnitude of the curvature. In 2D we use the normal curvature *κ***n** ≡ d**t**/d*s* where **t** is the unit tangent and *s* is a coordinate along the interface, measured positive in the direction of **t**. The stress can be integrated over a surface area in 3D or over an interface segment in 2D, around a given point **x**_*s*_(*t*) = (*x*_*s*_(*t*), *y*_*s*_(*t*)) on the interface, to obtain a local representation of the interfacial force term

14 $${{{{{{{{\bf{f}}}}}}}}}^{{{{{{{{\rm{I}}}}}}}}}({{{{{{{\bf{x}}}}}}}},t)={\lim }_{| {s}_{2}-{s}_{1}| \to 0}\int\nolimits_{{s}_{1}}^{{s}_{2}}{{{{{\rm{d}}}}}}s\ \gamma \frac{{{{{{\rm{d}}}}}}{{{{{{{\bf{t}}}}}}}}}{{{{{{\rm{ds}}}}}}}\ \delta [{{{{{{{\bf{x}}}}}}}}-{{{{{{{{\bf{x}}}}}}}}}_{s}(t)],$$

where *δ*[**x** − **x**_*s*_] = *δ*[*x* − *x*_*s*_]*δ*[*y* − *y*_*s*_] is the Dirac delta function in 2D. The segment from *s*_1_ to *s*_2_ is centered at the point **x**_*s*_(*t*) on the interface. However, the above representation is not quite useful for numerical implementation. For that the continuous interface is replaced by linearly connected discrete points *i* and an average value of the “point" force **f**^I^ in an Eulerian control volume *V* = Δ*x*Δ*y*, on which the **u** and **Q** equations are solved, is taken as

15 $$\frac{1}{V}\int\,{{{{{\rm{d}}}}}}V{{{{{{{{\bf{f}}}}}}}}}^{{{{{{{{\rm{I}}}}}}}}}=\frac{1}{V}\int\,{{d}}x{{d}}y\int\nolimits_{{s}_{1}}^{{s}_{2}}{{d}}s\,\gamma \frac{{{d}}{{{{{{{\bf{t}}}}}}}}}{{{d}}s}\,\delta [{{{{{{{\bf{x}}}}}}}}-{{{{{{{{\bf{x}}}}}}}}}_{s}(t)] \, \approx \, \frac{\gamma }{\Delta x\Delta y}{\sum }_{i=1}^{{N}_{f}}\Delta {{{{{{{{\bf{t}}}}}}}}}_{i},$$

where *N*_*f*_ are the number of front points enclosed in the Eulerian control volume *V* = Δ*x*Δ*y* and Δ**t**_*i*_ is the difference between the unit tangent pointing from *i* to *i* + 1 and the unit tangent pointing from *i* − 1 to *i*. The interfacial force is obviously zero for control volumes away from the interface or in which *N*_*f*_ = 0. Overall, the above force gives rise to a jump in stress across the interface. For detailed descriptions of the algorithm, the reader is referred to^64–66^. Also, the non-dimensionalization of Eq. (15) results in

16 $${f}_{o}^{{{{{{{{\rm{I}}}}}}}}}{{{{{{{{{\bf{f}}}}}}}}}^{{{{{{{{\rm{I}}}}}}}}}}^{{\prime} } \sim \frac{\gamma }{{x}_{o}^{2}}\frac{1}{\Delta {x}^{{\prime} }\Delta {y}^{{\prime} }}{\sum }_{i=1}^{{N}_{f}}\Delta {{{{{{{{\bf{t}}}}}}}}}_{i},$$

which provides a scale for the interfacial force term ${f}_{o}^{{{{{{{{\rm{I}}}}}}}}}=\gamma /{x}_{o}^{2}$ and therefore the non-dimensional parameter ${{{{{{{{\rm{Re}}}}}}}}}_{{{{{{{{\rm{I}}}}}}}}}$ in Eq. (12) turns out to be

17 $${{{{{{{{\rm{Re}}}}}}}}}_{{{{{{{{\rm{I}}}}}}}}}=\frac{{x}_{o}{t}_{o}{f}_{o}^{{{{{{{{\rm{I}}}}}}}}}}{{\eta }_{o}}=\frac{\gamma }{{\eta }_{o}{u}_{o}}=\frac{1}{{{{{{{{\rm{Ca}}}}}}}}},$$

the inverse of the capillary number Ca. Once all the terms are calculated, the equations for **u** and **Q** are integrated in time in a coupled manner using the pressure projection algorithm. The interface is then advected using the velocity field solution, and the algorithm moves to the next time step^64–66^. A feature of the present one-fluid formulation is that the anchoring conditions at the interface need not be input by hand. The advective fluxes in the continuum equations are reconstructed using a sixth-order weighted essentially non-oscillatory scheme^81,82^ which in present simulations has proven to reduce numerical diffusion and help conserve the droplet mass or area in 2D.

The extension of this method to flows with rigid bodies is not generally well posed in the rigid limit^83^. A different forcing term with coefficients to enforce the boundary conditions has been found to be effective in simulating flow past solid objects at moderate Reynold’s number and low Reynolds number turbulent flows^84^.

### Mean square displacement and VACF

To incorporate the memory effect in the stochastic force in the Langevin equation, let us start from an exponentially correlated random force

18 $$\langle F({t}^{{\prime} })F({t}^{{\prime\prime} })\rangle=\Lambda \exp [-| {t}^{{\prime} }-{t}^{{\prime\prime} }| /{\tau }_{F}],\ \langle F(t)\rangle=0,$$

where *τ*_*F*_ is the time scale which tells us the extent of the force memory, and Λ ≡ *Dα*^2^/*τ*_*F*_ with *D* and *α* being diffusion constant and coefficient of friction respectively. This effective Langevin description is equivalent to earlier approaches to describe passive tracers in active baths via generalized Langevin equations with instantaneous friction and noise with memory kernel^11,75–79^.

The equation of motion for a passive particle in such an active bath for the underdamped case can be written as

19 $$\dot{{{{{{{{\bf{r}}}}}}}}}={{{{{{{\bf{v}}}}}}}},\quad \dot{{{{{{{{\bf{v}}}}}}}}}=-\frac{\alpha }{m}{{{{{{{\bf{v}}}}}}}}+\frac{1}{m}{{{{{{{\bf{F}}}}}}}}(t)+\frac{\sqrt{2\alpha {k}_{B}{{{{{{{\mathcal{T}}}}}}}}}}{m}{{{{{{{\boldsymbol{\eta }}}}}}}}(t)=-\Gamma {{{{{{{\bf{v}}}}}}}}+\frac{1}{m}{{{{{{{\bf{F}}}}}}}}(t)+\sqrt{\frac{2\Gamma {k}_{B}{{{{{{{\mathcal{T}}}}}}}}}{m}} \, {{{{{{{\boldsymbol{\eta }}}}}}}}(t),$$

where Γ ≡ *α*/*m*, ${{{{{{{\mathcal{T}}}}}}}}$ is the temperature and ***η*** is a white noise with zero mean, unit variance, and flat power spectrum. For simplicity of the argument, we present results for two dimensions although the formulation can readily be extended to higher dimensions. The cross-correlation function 〈**v**(*t*) ⋅ **F**(*t*)〉 is in general non-zero and carries information about the balance between the energy supplied by the active bath to the droplet and the energy dissipated by the droplet due to the drag offered by the bath – the fluctuation-dissipation mechanism. This correlation function can be readily obtained from the expression for the velocity

20 $${{{{{{{\bf{v}}}}}}}}(t)={{{{{{{{\bf{v}}}}}}}}}_{0}{e}^{-\Gamma t}+{e}^{-\Gamma t}\int\nolimits_{0}^{t}{e}^{\Gamma {t}^{{\prime} }}\left(\frac{{{{{{{{\bf{F}}}}}}}}({t}^{{\prime} })}{m}+\sqrt{\frac{2\Gamma {k}_{B}{{{{{{{\mathcal{T}}}}}}}}}{m}}{{{{{{{\boldsymbol{\eta }}}}}}}}({t}^{{\prime} })\right){{{{{\rm{d}}}}}}{t}^{{\prime} }.$$

Using this we obtain in the steady state limit,

21 $$\langle {{{{{{{\bf{v}}}}}}}}(t)\cdot {{{{{{{\bf{F}}}}}}}}(t)\rangle=\frac{2\Lambda {\tau }_{F}\Gamma }{m\Gamma (1+{\tau }_{F}\Gamma )}.$$

For *τ*_*F *_Γ ≫ 1, i.e. *τ*_*F*_ ≫ 1/Γ = *m*/*α* = *τ*_*m*_, we have

22 $$\begin{array}{r}\langle {{{{{{{\bf{v}}}}}}}}(t)\cdot {{{{{{{\bf{F}}}}}}}}(t)\rangle=\frac{2\Lambda }{m\Gamma }.\end{array}$$

For the athermal case, i.e. when ${{{{{{{\mathcal{T}}}}}}}}\to 0$, the steady state velocity auto-correlation function (VACF) can also be calculated as

23 $$\begin{array}{r}\langle {{{{{{{\bf{v}}}}}}}}(t)\cdot {{{{{{{\bf{v}}}}}}}}(0)\rangle=\frac{2\Lambda {\tau }_{F}}{{\alpha }^{2}}\left[\frac{{\tau }_{m}{e}^{-t/{\tau }_{m}}-{\tau }_{F}{e}^{-t/{\tau }_{F}}}{{{\tau }_{m}}^{2}-{{\tau }_{F}}^{2}}\right]=2D\left[\frac{{\tau }_{m}{e}^{-t/{\tau }_{m}}-{\tau }_{F}{e}^{-t/{\tau }_{F}}}{{{\tau }_{m}}^{2}-{{\tau }_{F}}^{2}}\right]\end{array}$$

Therefore, we have two competing time scales: the persistence time *τ*_*F*_ and the inertial time *τ*_*m*_. For *τ*_*F*_ ≪ *τ*_*m*_,

24 $$\begin{array}{r}\langle {{{{{{{\bf{v}}}}}}}}(t)\cdot {{{{{{{\bf{v}}}}}}}}(0)\rangle=\frac{2D}{{\tau }_{m}}\exp [-t/{\tau }_{m}]\end{array}$$

while for *τ*_*F*_ ≫ *τ*_*m*_,

25 $$\begin{array}{r}\langle {{{{{{{\bf{v}}}}}}}}(t)\cdot {{{{{{{\bf{v}}}}}}}}(0)\rangle=\frac{2D}{{\tau }_{F}}\exp [-t/{\tau }_{F}].\end{array}$$

The mean squared displacement (MSD) can be calculated from the VACF as $\langle | {{{{{{{\bf{r}}}}}}}}(t)-{{{{{{{{\bf{r}}}}}}}}}_{0}{| }^{2}\rangle=2\int\nolimits_{0}^{t}d{t}_{1}\int\nolimits_{0}^{{t}_{1}}d{t}_{2}\langle {{{{{{{\bf{v}}}}}}}}({t}_{1})\cdot {{{{{{{\bf{v}}}}}}}}({t}_{2})\rangle$. In the limit of *τ*_*F*_ ≫ *τ*_*m*_ and for a zero initial velocity of the particle, the approximate form of the mean square displacement becomes

26 $$\begin{array}{r}\langle | {{{{{{{\bf{r}}}}}}}}(t)-{{{{{{{{\bf{r}}}}}}}}}_{0}{| }^{2}\rangle \, \approx \, 4D{\tau }_{F}\left({e}^{-\frac{t}{{\tau }_{F}}}-1+\frac{t}{{\tau }_{F}}\right)-8D{\tau }_{m}\left({e}^{-\frac{t}{{\tau }_{m}}}-1\right)\left({e}^{-\frac{t}{{\tau }_{F}}}-1\right).\end{array}$$

This solution is shown in Fig. 3 (b, inset).

To make the connection to the effect of size on the velocity of the droplet, we note that the mean squared velocity in the long time limit for the athermal case can be obtained as

27 $$\begin{array}{r}\langle {v}^{2}\rangle=\frac{2\Lambda }{{\alpha }^{2}}\frac{{\tau }_{F}\Gamma }{1+{\tau }_{F}\Gamma }=\frac{2D}{{\tau }_{m}+{\tau }_{F}}=\frac{2D/{\tau }_{F}}{1+m/\alpha {\tau }_{F}}.\end{array}$$

If we identify $2D/{\tau }_{F}={u}_{0}^{2}$ where *u*_0_ is some active velocity of the particle, then it is clear that particles with larger radius ( ≡ *m*) will have smaller velocities than the smaller particles. In Supplementary Fig. 4, we show that the ensemble averaged trajectory length of the particle over a time period, which gives the average velocity, decreases as ~ 1/*R*.

### Supplementary information

Supplementary Information

Peer Review File

## Data Availability

The raw simulation data will be available upon request in a hard drive because of its large size (TB). The processed data of the plots are available as Source Data files in the Figshare database [10.6084/m9.figshare.25417522].

## Code availability

The custom computer codes used for the simulations are available from the authors upon request.
