# Supplementary material for: Anomalous dynamics of a passive droplet in active turbulence
Source: Nat Commun. 2024 May 2;15:3704. doi: 10.1038/s41467-024-47727-1 (PMC11066042; doi:10.1038/s41467-024-47727-1)
Supplement: Supplementary file 1 — Supplementary Information [file 41467_2024_47727_MOESM1_ESM.pdf]

Supplemental Material: Anomalous Dynamics of a Passive Droplet in Active Turbulence

Chamkor Singh*
Department of Physics, Central University of Punjab, Bathinda 151401, India

Abhishek Chaudhuri†
Department of Physical Sciences, Indian Institute of Science Education and Research Mohali, Manauli 140306, India

(Dated: April 9, 2024)

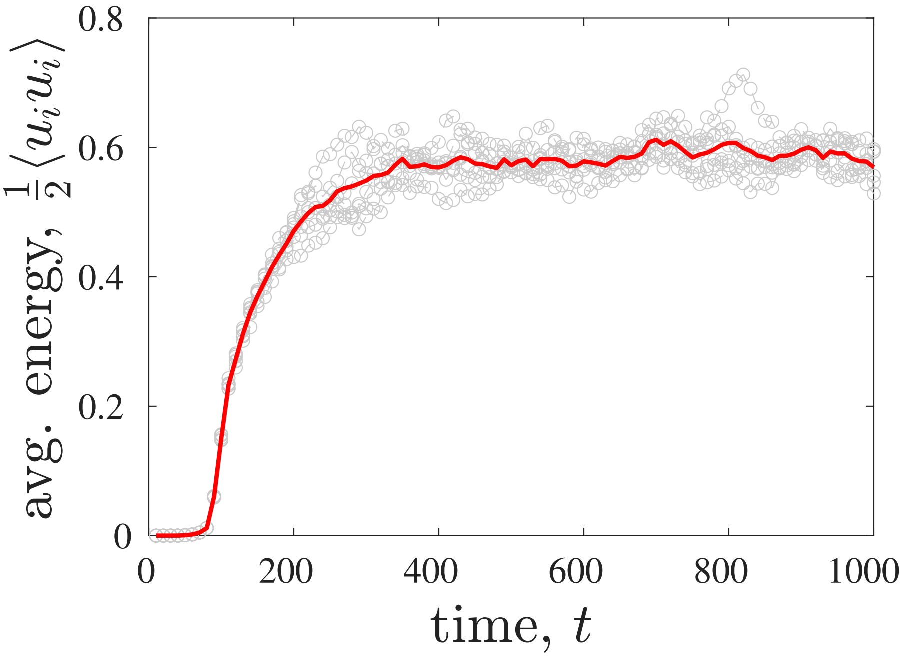

Supplementary Figure 1: Average kinetic energy per unit mass with time. Gray symbols for individual realizations and the red line is ensemble average. Time from $t = 0$ to $t \approx 300$, when the active turbulence is still developing, is neglected and a time window of $300 \leq t \leq 1000$ is considered for the statistical results presented in this study.

* chamkor.singh@cup.edu.in
† abhishek@iisermohali.ac.in

[1] L. Giomi, Geometry and topology of turbulence in active nematics, Physical Review X **5**, 031003 (2015).
[2] B. Martínez-Prat, R. Alert, F. Meng, J. Ignés-Mullol, J.-F. Joanny, J. Casademunt, R. Golestanian, and F. Sagués, Scaling regimes of active turbulence with external dissipation, Physical Review X **11**, 031065 (2021).

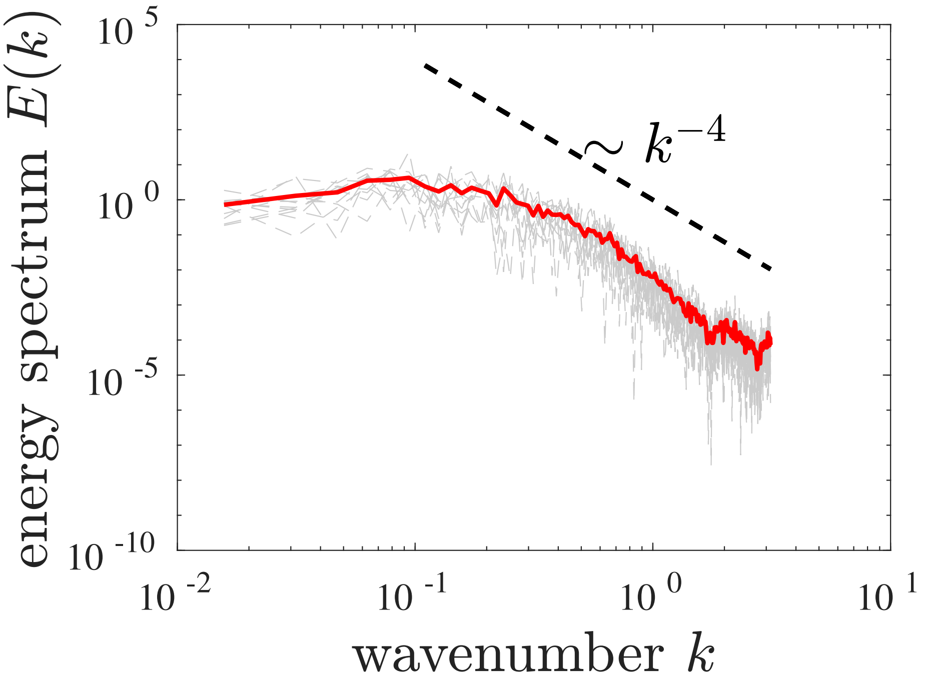

Supplementary Figure 2: The energy spectrum $E(k)$ of the active bath with spectral exponent close to -4, a behavior which has been observed recently [1, 2].

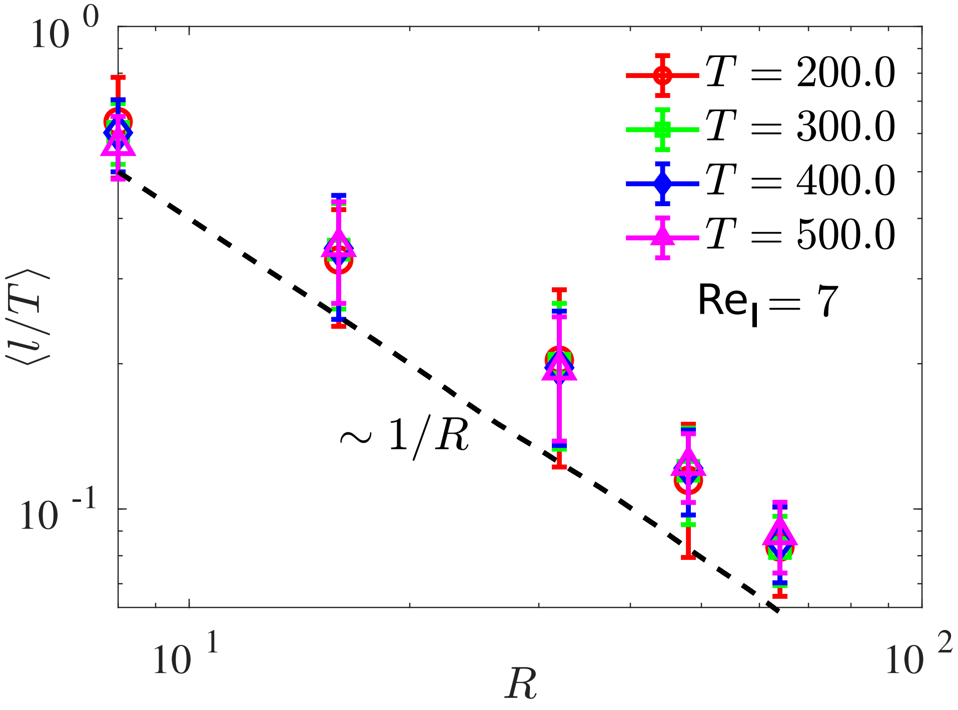

Supplementary Figure 3: Ensemble averaged total length l of trajectory divided by the time period of the trajectory T, with varying T and R.

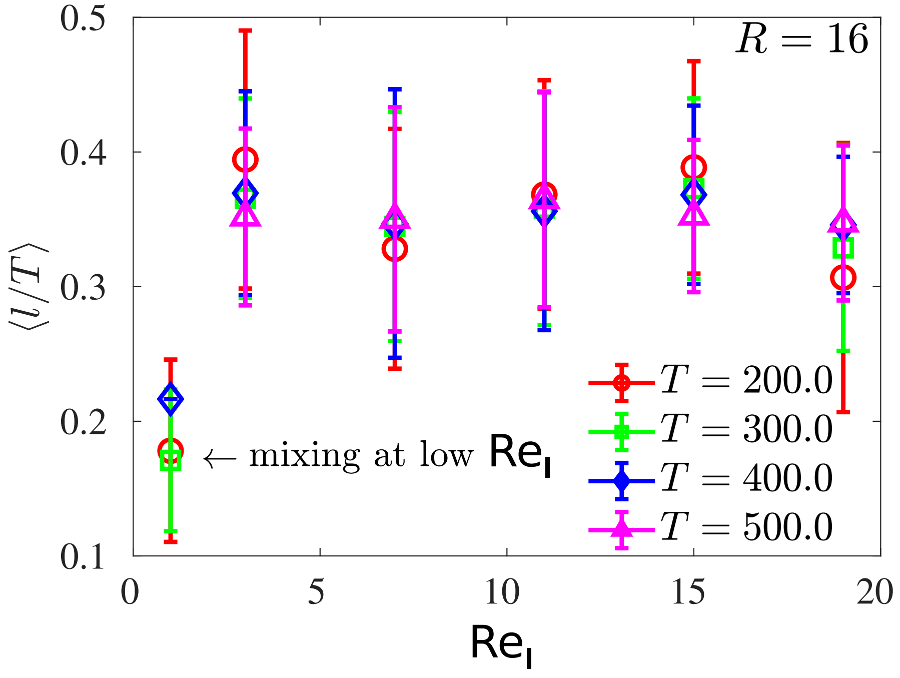

Supplementary Figure 4: Ensemble averaged total length l of trajectory divided by the time period of the trajectory T, with varying T and $\mathrm{Re_I}$.
