# Supplementary material for: Anomalous dynamics of a passive droplet in active turbulence
Source: Nat Commun. 2024 May 2;15:3704. doi: 10.1038/s41467-024-47727-1 (PMC11066042; doi:10.1038/s41467-024-47727-1)
Supplement: Supplementary file 2 — Peer Review File [file 41467_2024_47727_MOESM2_ESM.pdf]

nature portfolio

Peer Review File

Anomalous Dynamics of a Passive Droplet in Active Turbulence

Open Access This file is licensed under a Creative Commons Attribution 4.0 International License, which permits use, sharing, adaptation, distribution and reproduction in any medium or format, as long as you give appropriate credit to the original author(s) and the source, provide a link to the Creative Commons license, and indicate if changes were made. In the cases where the authors are anonymous, such as is the case for the reports of anonymous peer reviewers, author attribution should be to 'Anonymous Referee' followed by a clear attribution to the source work. The images or other third party material in this file are included in the article's Creative Commons license, unless indicated otherwise in a credit line to the material. If material is not included in the article's Creative Commons license and your intended use is not permitted by statutory regulation or exceeds the permitted use, you will need to obtain permission directly from the copyright holder. To view a copy of this license, visit http://creativecommons.org/licenses/by/4.0/.

REVIEWER COMMENTS

Reviewer #1 (Remarks to the Author):

Motivated by recent experimental work at Dogic's Lab, Singh and Chaudhuri in their manuscript approach the problem of studying the motion of a passive droplet suspended in an anisotropic nematic active fluid. To this goal, they consider a hydrodynamic continuous approach where the liquid crystal dynamics is governed by the Beris-Edwards equation, accounting for the elastic relaxation of the liquid crystal and the advection of the underlying fluid, while the fluid itself follows the viscous incompressible Navier-Stokes equation featuring stress contributions due to liquid crystalline elasticity and an active contribution σ^A, widely used in the literature to model non-equilibrium energy injection in active nematics.

The authors set the parameters of their simulations to enter the chaotic active turbulent phase and analyze the droplet's behavior at varying the droplet size, through a series of measurements (spectral analysis, translational and angular displacement distribution and autocorrelation). The main result of the study is that droplet's dynamics may feature a crossover between superdiffusive to diffusive regime, depending on the size of the suspended passive particle. Finally, the authors discuss how the droplet's motion can be qualitatively described by a Langevin model with exponentially correlated random forces.

While the idea beyond this study is very interesting and timely, however the manuscript in its present form seems uncomplete with important details omitted. Some of the conclusions of the paper are drawn by looking at only a very limited dataset and therefore lack robustness and the validity of the model for the case under consideration remains questionable. Overall, I believe that the manuscript needs considerable revision and more supporting data. In the following I provide a detailed report of my perplexities which I believe should be thoroughly addressed before considering this manuscript further.

1. The authors suspend a passive droplet in an active nematics but the inner structure of the droplet is not specified. Is the droplet isotropic or does it contain a passive nematic? The explicit expression of the molecular field H in the model section suggests that the liquid crystalline phase is not confined outside of the droplet but it is also present inside the droplet, i.e. Q≠0 everywhere. Therefore, looking at the expression of the active stress tensor σ^A=-ζQ which means that the droplet is active and not passive as the authors state in the text. If the droplet is isotropic, i.e. Q=0, the expression of H cannot be the one reported in the methods.

2. How is the droplet modeled? No detail is provided in the methods section, not even in the section “Droplet interfacial dynamics”. The authors state that “the droplet is suspended as a second phase in the active medium”. Shall I understand the authors simulate a phase field which defines the droplet? What is its dynamics?

3. In the same section the authors state that one feature of the model is that anchoring conditions emerge from the solution itself. What are these resulting anchoring conditions? Is it homeotropic, or tangential? Is it an equilibrium or active feature? Previous work (see for instance Phys. Rev. Lett. 113, 248303) has demonstrated that activity can induce anchoring at the droplet interface. Does the model of the authors reproduce these features. The authors should provide evidence.

4. What does it mean that the sixth order scheme used to reconstruct the advective fluxes is 'quite' volume conservative?

5. There exist two main notations prevalent in the literature, one defining the active stress as $\sigma^A=-\zeta Q$, and the other as $\sigma^A=\alpha Q$. Extensile activity corresponds to $\zeta>0,\alpha<0$ and contractile activity to $\zeta<0,\alpha>0$. The authors should choose one of these two notations to avoid confusion.

6. Nowhere in the main text is reported what kind of activity is chosen. The reader needs to look for this (relevant) information by itself in table I where a value for the dimensionless activity is provided. Far from being semantic, this is a relevant point as the effect of extensile/contractile activity sensibly affects the elastic instability of a liquid crystal.

7. The authors state that “as a general property, the system of equations is unstable around the state $Q=0,u=0$”. This is not true. The equations that the authors consider become unstable when activity is increased beyond a certain critical value which depends on the size of the system.

8. Why there is no stress contribution due to droplet’s interface in the Navier-Stokes equation? This is unphysical. And even more importantly as the authors study droplet’s deformations. Interfaces exert a stress on the surrounding fluid generating a flow to restore spherical shapes. This contribution being neglected invalidates the results presented in Fig. 2c.

9. Coming to the main text, in the introduction the authors state that theoretical studies of passive tracers either use a generalized Langevin approach or particle based simulations. The authors are probably not aware of a series of works by Marenduzzo and Gonnella where a hydrodynamics model

for microphase separation was used to approach the problem, see for instance Sci Rep 9, 2801 (2019).

10. The kinetic energy spectrum should be computed as an ensemble average over uncorrelated configurations and not as a time average. Moreover, the energy spectrum of a turbulent active nematic is something very well known in the literature. The authors may consider removing panel f from Fig. 1 and present it as supplementary information. Analogous suggestion for panel Fig. 1e.

11. Vertical and horizontal span in the definition of parameter A(t) measuring droplet deformations sensibly depend on the reference system. Why not using a parameter measuring the sphericity of the droplet, independently of the reference system?

12. The authors claim that the spectral plots in Fig. 2d exhibit nearly $1/\omega$ decay. I am not convinced. Can the authors provide fit with errors? Analogous considerations apply for other scaling laws reported in Fig. 2e,f.

13. How are the spectra $S(\omega)$ defined? On a related notice, the quantities $\omega_{\Delta r}$,$\omega_{\Delta\theta}$,ω_A in Fig. 2d,e,f are also undefined. I believe the authors mean that ω is the Fourier frequency, which should not be labelled, while subscripts should be used to label the corresponding spectra $S_{\Delta r}$,$S_{\Delta\theta}$,S_A.

14. At line 150, what is d(r)? I could not find its definition anywhere.

15. In Fig. 2a-c on the x-axis is t the dimensional or dimensionless time? It would be advisable to compare the time normalized by the active timescale ζ/η. Analogously the y-axis in Fig. 1a could be normalized with respect to the active lengthscale $\sqrt{K/\zeta}$.

16. The author claim that Fig. 3a shows the existence of a diffusive regime, but I cannot see it from the figure. Could the author provide evidence?

17. I am also concerned about the fact that the authors considered only 3 values for the droplet radius. I believe that more values should be considered in order to draw significant conclusions.

18. In the conclusions the authors claim that the transition from the superdiffusive to the diffusive regime also depend on the properties of the active bath. However, from what what reported in the manuscript and in Table I the authors only varied the droplet's radius. Indeed, it would be interesting to test the behavior of the system at varying the activity considering both extensile and contractile activity. This is a point that an article on the anomalous dynamics of a passive droplet in active turbulence should not neglect.

Reviewer #2 (Remarks to the Author):

The dynamics of a passive droplet suspended in active nematics subjected to friction is studied in this manuscript and the effect of droplet size is analysed. It is observed that the droplet moves almost ballistically for longer time scales and that it moves faster for smaller radii. The problem is interesting and could help explain phenomena in biological systems such as the motion of tracers and other particles in bacterial swarms. However, in the opinion of this referee, this work should be submitted to a more specialised journal after some revision and verification of the results (suggestions below). Many previous works have investigated the dynamics of droplets in active nematics with some of them being acknowledged in the introduction. For instance, in [Soft Matter, 10, 7826-7837(2014)] it was found that the passive droplets might acquire directed motion due to the formation of topological defects close to the interface (do you observe them in your simulations?). Other works have studied the dynamics of active droplets in a passive fluid [e.g.: Nat. Phys. 17, 260–266 (2021), Phys. Rev. Research 5, 033165 (2023), Soft Matter, 13, 5933-5941 (2017)]. Thus, the present study is original to the best of my knowledge but is an extension of previous works in the literature.

Regarding the results, the MSD in Fig. 3b should be double-checked. This ballistic behaviour could be an indication of finite-size effects. Because you simulated a relatively small system (200X200 or 400X400) the active nematics could be forming directed flows due to the periodic boundary conditions instead of random vortices. The authors should check if this ballistic regime persists for different system sizes (or frictions) and with a closed system instead of periodic ones.

It is not completely clear that the energy spectrum in fig. 1f follows a -4 power law at the end. Anyway, the authors should also check if the -1 and +1 power laws for small and intermediate wave numbers are found. Depending on the friction, other power laws can be observed [Soft Matter, 16, 4256-4266 (2020)].

What is the method used to solve the equations for the dynamics (Navier-Stokes and Beris-Edwards)?

It is said that the droplet follows “stays” and “runs”. Is it the same as the Levy walks observed in [Phys. Rev. Lett. 127, 118001]?

Reviewer #3 (Remarks to the Author):

This theoretical study investigates the dynamics of a passive deformable droplet in active nematic turbulence described by the standard partial differential equations for active nematics. The droplet exhibits fluctuating deformations and movement. Here, the mean-square displacement shows a superdiffusive regime that transitions to diffusive behavior for large time scales. The main result of the study is that the radius is very important in this context. When it is smaller than the correlation length of the velocity field, the droplet experiences strong advection, whereas when it is larger, the droplet will remain more localized. The authors end the manuscript with an effective Langevin description of the droplet motion that qualitatively captures the temporal correlations observed.

Overall, the manuscript is well written and the data is presented in great detail. The main result concerning the droplet size compared to spatial correlations of the surrounding flow field is indeed very interesting. To the best of my knowledge, it has not been explored before how the motion of passive inclusions in active turbulence is influenced by the interplay of spatial correlations of the flow and the size of the inclusions. I expect that this study might inspire experiments in the research field and further theoretical investigations.

However, there are a few key points that in my opinion should be addressed before publication. These concern the actual role of the deformability of the droplet, the correspondence to the effective Langevin description and overall the limited data set varying only one parameter. Therefore, I would recommend publication in a more specialized journal.

In the following, I explain my main concerns and comments that should be addressed before publication.

Major comments

1. The authors stress that the deformability of the droplet is taken into account. However, they never really address the impact of deformability on their results. They mostly show how the size of the droplet changes its motion, which is a very interesting result in its own right. But I wonder whether the same effect would be observed when looking at a rigid inclusion. What happens if the deformability (interfacial forces) is varied?

2. The authors often talk about the transition from the superdiffusive to the diffusive regime without ever really showing the transition in the MSD in the figures. At least, a line showing diffusive behavior ~(t-t_0) should be added to Fig. 3 (b). If the transition is not really visible in a clear way, it might be advisable to increase the length of the simulations and the number of times the simulations are repeated (15 times seems hardly enough to calculate an MSD). If this is not feasible for computational reasons, the authors should justify such a low number of repetitions.

3. Concerning the effective Langevin description, the authors should state where their approach differs from earlier approaches to describe passive tracers in active baths or if it is indeed equivalent to these earlier approaches, in particular concerning the results derived in the Methods section. Tracers in active baths have been modeled before via generalized Langevin equations with instantaneous friction and noise with memory kernel, see for example Wu, Libchaber, Phys. Rev. Lett. 84, 3017 (2000) or Maggi, Paoluzzi, Pellicciotta, Lepore, Angelani, Di Leonardo, Phys. Rev. Lett. 113, 238303 (2014). Further, the dynamics of active particles themselves have been modeled in a similar way, see for example Bechinger, Di Leonardo, Löwen, Reichhardt, Volpe, Volpe, Rev. Mod. Phys. 88, 045006 (2016). The memory is sometimes assumed to be generated by an Ornstein-Uhlenbeck process [G. E. Uhlenbeck, L. S. Ornstein, Phys. Rev. 36, 823 (1930)]. Is the approach used by the authors equivalent to these earlier approaches? If yes, this point should be mentioned and references should be given.

4. To my understanding, the effective Langevin description can only qualitatively reproduce some aspects of the droplet motion, however, it has no predictive power concerning the effect of the droplet radius as the radius might influence many of the coefficients in the effective Langevin description. Thus, in the current form the Langevin model does not explain the most interesting aspect of the observations. Here, it should be stated more clearly what the benefit is of introducing the effective description at all.

To increase the significance of the effective description, might it be possible to map the dynamics obtained from the numerical calculations to the effective Langevin model? For example, can an effective temperature be calculated? In my opinion, this would be an interesting point because effective temperatures have been calculated for active systems before and usually increase with increasing activity. What is expected in the present system? How does the radius of the droplet impact the effective temperature?

5. Why is the absolute step size used when calculating the step autocorrelation function and not the step vector? Step sizes with the same magnitude but opposite direction are thus perfectly correlated. I understand that the angular steps are also defined and cover this information. However, the correlations of these angular steps are never determined. My main concern here comes from the fact that the step autocorrelation time is later compared to the velocity autocorrelation time obtained via the effective Langevin dynamics, which is not calculated from the absolute velocity but from the velocity vector.

6. The authors should cite theoretical investigations of tracer particles in active fluids such as bacterial suspensions described by continuum-theoretical models. In particular, there are a few works that investigate the impact of flow patterns in active turbulence on tracer dynamics: Sanjay, Joy, Phys. Rev. Fluids 5, 024302 (2020), Mukherjee, Singh, James, Ray, Phys. Rev. Lett. 127, 118001 (2021), Reinken, Klapp, Wilczek, Phys. Rev. Fluids 7, 084501 (2022). These should be cited, e.g., in l. 38.

Further, there are theoretical attempts to justify the superdiffusive regime observed in bacterial turbulence, which should be cited in ll. 154F: Ariel, Be'er, Reynolds, Phys. Rev. Lett. 118, 228102 (2017).

Minor comments:

1. The authors use an underdamped Langevin equation for the effective description. I wonder if an overdamped description would not be sufficient in this case. Is the inertia of the droplet really important here? Taking an overdamped description would significantly simplify the calculations in the Methods section. The reduced number of independent parameters might also help to find a better correspondence between the observations from the numerical simulations and the effective Langevin description.

2. The introduction of the initial state is somewhat confusing (l. 91). Is the initial state isotropic or nematic?

3. A more detailed introduction to how the interfacial forces are calculated in Eq.(4) is needed to really understand the model. This should be given in the Methods section.

4. What is the significance of Fig. 3 (a)? Can the behavior in the MSD for smaller values of time lag only be observed because the point t_0 here is chosen as the beginning of the simulation when

turbulence has not developed yet? If this is the case, I would not show this plot and only show (b) where t_0 is chosen in the fully developed turbulent state.

5. I think it is not necessary to show Fig. 3 (f). This could be shifted to a Supplemental Material or not shown at all. Not showing Fig. (a) and (f) would also allow to increase the size of the other figures, which are too small in my opinion.

6. Similarly, I would suggest to split Fig. 1 up into two figures to increase the size and readability.

7. It should be better justified why \tau_{\Delta r} obtained from the step autocorrelation function in the simulation corresponds to the correlation time obtained via the velocity correlation function in the effective Langevin description (see ll. 186ff). After all, one quantity characterizes correlations in the step size, the other velocity correlations.

8. I think it would benefit the manuscript if the fluctuation-dissipation theorem and equilibrium versus non-equilibrium is discussed in the context of the effective Langevin description. My understanding is that the FDT is not assumed to hold from the outset (Eqs.(14) and (15)), which is indeed not a necessity as we are dealing with a non-equilibrium system. However, an effective temperature is introduced between Eqs.(20) and (21). Does this introduce an effective equilibrium assumption? I suggest discussing this point in the main text around l. 186 and in the Methods section around l. 310.

9. The introduction of the model equations and the rescaling procedure is shifted to the Methods section, which helps in keeping the manuscript accessible. However, in l. 110, when the explored range for the radii of the droplet is given, there has to be some kind of scale given as well. What does a radius of 15 mean? Relative to what? At least in the case of the radius the scaling has to be discussed briefly in the main text as well.

10. The sentence in ll. 142ff is confusing. Is it supposed to mean “... have dynamics distinct from the motion increments.”?

RESPONSE TO REVIEW REPORT: MANUSCRIPT NCOMMS-23-41742

Summary of main revisions in the manuscript:

We have now performed two additional set of simulations and analyzed data for (1) five additional droplet radii (R), and (2) varying the interfacial tension – a total of 2x140=280 simulations. Also in all the new simulations we increased the system size to 450x450 with grid size 576x576 keeping the overall simulation time manageable. This we believe has helped to mitigate, the boundary effects on the droplet trajectories to get better data for mean square displacement (MSD), velocity auto-correlation function (VACF) etc. We have managed to reach simulation time > 1000 for each new simulation and now the transition from the ballistic to diffusive or superdiffusive regimes is quite clear. The ensemble average is also improved – now it is over double the number of realizations we had previously for each curve in MSD, VACF, and other results. Instead of computing the step size autocorrelation function, we now compute the VACF, after fruitful suggestion by one of the referees, and we see that it has given us additional insights how the dynamics of droplet trajectories are affected in an active bath.

We have added new results with interfacial tension variation in new Fig. 4: While varying surface tension does not significantly change the VACF and MSD, or the transition time of MSD, we show that upon significantly lowering the surface tension, the droplet begins to break and mix with the active bath. The ensemble averaged total length of trajectory in a given period comes out to be inversely proportional to the radius R which endorses our claim that smaller droplets travel longer distances.

We feel that we have addressed the concerns of the reviewers and significantly improved the manuscript's quality with (i) improved ensemble averaging, (ii) robustness of our results for a wide range of radii, (iii) additional data demonstrating the effects of varying surface tension on the deformability of the droplet and its dynamics, (iv) improved MSD statistics, and (v) new and significant results about VACF.

Below we provide point by point response to the referee comments.

Reviewer #1 (Remarks to the Author):

Motivated by recent experimental work at Dogic's Lab, Singh and Chaudhuri in their manuscript approach the problem of studying the motion of a passive droplet suspended in an anisotropic nematic active fluid. To this goal, they consider a hydrodynamic continuous approach where the liquid crystal dynamics is governed by the Beris-Edwards equation, accounting for the elastic relaxation of the liquid crystal and the advection of the underlying fluid, while the fluid itself follows the viscous incompressible Navier-Stokes equation featuring stress contributions due to liquid crystalline elasticity and an active contribution σ^A, widely used in the literature to model non-equilibrium energy injection in active nematics.

The authors set the parameters of their simulations to enter the chaotic active turbulent phase and analyze the droplet's behavior at varying the droplet size, through a series of measurements (spectral analysis, translational and angular displacement distribution and autocorrelation). The main result of the study is that droplet's dynamics may feature a crossover between superdiffusive to diffusive regime, depending on the size of the suspended passive particle. Finally, the authors discuss how the droplet's motion can be qualitatively described by a Langevin model with exponentially correlated random forces.

While the idea beyond this study is very interesting and timely, however the manuscript in its present form seems uncomplete with important details omitted. Some of the conclusions of the paper are drawn by looking at only a very limited dataset and therefore lack robustness and the validity of the model for the case under consideration remains questionable. Overall, I believe that the manuscript needs considerable revision and more supporting data. In the following I provide a detailed report of my perplexities which I believe should be thoroughly addressed before considering this manuscript further.

We thank the reviewer for considering the study to be interesting and timely. We are pleased to report that we have now carried out a number of lengthy simulations and examined the data in order to persuasively address the reviewer's concerns about the small data set and the model's lack of validity and robustness for the instance under discussion. We now report results for (i) five more sets of droplet radius and (ii) for a set of surface tension values in the revised text. In addition, we have increased the system size while keeping the overall simulation time manageable. Further, we have managed to reach simulation time > 10^3 for each parameter variation. With this large dataset and the ensuing analysis that amply illustrates the reliability of our findings, we genuinely hope the reviewer would deem the present draft of the work acceptable for publication.

- Below we provide a detailed point by point response to the reviewer comments.

1. The authors suspend a passive droplet in an active nematics but the inner structure of the droplet is not specified. Is the droplet isotropic or does it contain a passive nematic? The explicit expression of the molecular field H in the model section suggests that the liquid crystalline phase is not confined outside of the droplet but it is also present inside the droplet, i.e. $Q \neq 0$ everywhere. Therefore, looking at the expression of the active stress tensor $\sigma^A = -\zeta Q$

which means that the droplet is active and not passive as the authors state in the text. If the droplet is isotropic, i.e. Q=0, the expression of H cannot be the one reported in the methods.

- We are sorry for this confusion. In our study, the droplet contains a passive nematic. The liquid crystalline phase is present inside the droplet as well. We distinguish the environment inside and outside the droplet using the active stress tensor $\sigma^A = -\zeta Q$. Inside the droplet, $\zeta = 0$ while outside $\zeta \neq 0$. Further, the density and viscosity inside the droplet is twice that of the outer bath (see Table I for the parameter values). We have now made corrections to the manuscript to clarify this aspect. As the droplet moves, the activity, density and viscosity fields are updated accordingly. The details about the droplet simulation and its dynamics are now addressed in response to follow up questions by the reviewer (see below). We have now clarified this point in the manuscript.

2. How is the droplet modeled? No detail is provided in the methods section, not even in the section "Droplet interfacial dynamics". The authors state that "the droplet is suspended as a second phase in the active medium". Shall I understand the authors simulate a phase field which defines the droplet? What is its dynamics?

- We are sorry that the droplet model could not be made clearer by the material we had supplied in the text. The droplet is modeled using a front tracking technique, which is widely used to simulate unstable multi-fluid flows where incompressible fluids with varying densities and viscosities are separated by a sharp interface or front (see S. O. Unverdi and T. Tryggvason J. Comp. Phys. (1992), ref 60 in the manuscript). In this depiction, the interface is explicitly represented by an independent, unstructured grid for the fluid flow that passes through the stationary grid. As the calculations proceed, the interface's grid needs to be reorganized due to its ongoing deformation. Surface tension effects can be naturally incorporated into the tracked interface while preserving a distinct density and viscosity stratification. We now go into more detail about this in the section on Methods.

3. In the same section the authors state that one feature of the model is that anchoring conditions emerge from the solution itself. What are these resulting anchoring conditions? Is it homeotropic, or tangential? Is it an equilibrium or active feature? Previous work (see for instance Phys. Rev. Lett. 113, 248303) has demonstrated that activity can induce anchoring at the droplet interface. Does the model of the authors reproduce these features. The authors should provide evidence.

- We apologize once more for any confusion. We agree that the statement is vague and we have removed it from the current version of the manuscript. We meant that in the present method, we do not need to explicitly fix anchoring conditions at the interface of the droplet as it moves. We would like to emphasize that our method of inclusion of the droplet is robust and has been shown previously to be excellent in understanding bubble dynamics both in two and three dimensions. The primary advantage of this approach is that interfaces can interact in a rather natural way, since the gradients in properties across the interface simply add or cancel as the grid distribution is constructed from the information carried by the tracked front (see R. Mittal and G. Iaccarino, Ann. Rev. Fluid Mech. (2005) and

references therein). In order to look at anchoring at the droplet interface, we could have looked at the Q-tensor field solution near the interface. However, we have not pursued to check this as this would have altered the broader objectives of the study, i.e. the effect of deformability of the droplet and the droplet size on its dynamics in an active turbulent medium. We would like to point out that we have the data of the Q-tensor filed as a function of time and space and we would be happy to share it.

4. What does it mean that the sixth order scheme used to reconstruct the advective fluxes is 'quite' volume conservative?

- We meant that as the solution proceeds in time, the droplet volume (in 2D, it is the area occupied by the droplet) remains constant and there are negligible numerical errors leading to mass loss or gain of the droplet. We have now rephrased this statement in the revised manuscript to remove any confusion.

5. There exist two main notations prevalent in the literature, one defining the active stress as σ^A=-ζQ, and the other as σ^A=αQ. Extensile activity corresponds to ζ>0,α<0 and contractile activity to ζ<0,α>0. The authors should choose one of these two notations to avoid confusion.

- We thank the reviewer for pointing this out. In the current manuscript we have used the first notation everywhere, i.e. $\sigma^A = -\zeta Q$. We have also explicity mentioned in the revised manuscript that we are only looking at extensile systems $\zeta > 0$ in this study.

6. Nowhere in the main text is reported what kind of activity is chosen. The reader needs to look for this (relevant) information by itself in table I where a value for the dimensionless activity is provided. Far from being semantic, this is a relevant point as the effect of extensile/contractile activity sensibly affects the elastic instability of a liquid crystal.

- As mentioned in the previous response we have now corrected this in our revised manuscript.

7. The authors state that "as a general property, the system of equations is unstable around the state Q=0,u=0". This is not true. The equations that the authors consider become unstable when activity is increased beyond a certain critical value which depends on the size of the system.

- We completely agree that the equations become unstable when activity is increased beyond a certain critical value dependent on the system size and other model parameters. We have now made this point clear in the revised manuscript and have rephrased the previously incorrect statement.

8. Why there is no stress contribution due to droplet's interface in the Navier-Stokes equation? This is unphysical. And even more importantly as the authors study droplet's deformations. Interfaces exert a stress on the surrounding fluid generating a flow to restore

spherical shapes. This contribution being neglected invalidates the results presented in Fig. 2c.

- We regret this confusion due to our presentation of the numerical equations. There is indeed a stress contribution due to surface tension in our system – the term which we had denoted by f^I in the manuscript (Methods section, Eq. 4). This is in principle equal to the divergence of the interfacial stress. Although the term existed in the previous manuscript, we had not given a detailed description about it, naively assuming a familiarity with the formalism (S. O. Unverdi and T. Tryggvason J. Comp. Phys. (1992)). This interfacial force term acts singularly at the interface. It is therefore introduced as a localized forcing term in the momentum equations. The location of the interface does not generally coincide with the nodes of the Cartesian grid. The forcing is therefore distributes over a band of cells around the material point and this distributed force is incorporated on the momentum equation. The sharp delta function used in the defintion of f^I is essentially replaced by a smoother distribution function suitable for use on a discrete mesh.

- Please note that the boundary conditions at the interface are not explicitly needed in the numerical formulation we have used. However, for the sake of completeness, the tangential component of the total stress and the velocity fields are continuous while the normal component of the total stress is discontinous. In the revised manuscript, we have provided detailed description of the same.

9. Coming to the main text, in the introduction the authors state that theoretical studies of passive tracers either use a generalized Langevin approach or particle based simulations. The authors are probably not aware of a series of works by Marenduzzo and Gonnella where a hydrodynamics model for microphase separation was used to approach the problem, see for instance Sci Rep 9, 2801 (2019).

- We thank the referee for the making us aware of the above study. We have now corrected our statement and cited the reference in the appropriate context.

10. The kinetic energy spectrum should be computed as an ensemble average over uncorrelated configurations and not as a time average. Moreover, the energy spectrum of a turbulent active nematic is something very well known in the literature. The authors may consider removing panel f from Fig. 1 and present it as supplementary information. Analogous suggestion for panel Fig. 1e.

- We thank the referee for the valuable suggestion. In our simulations for the active fluid without the droplet, the configurations that we choose for a given initial condition were well separated in time assuming that they are decorrelated. With the expensive nature of the simulations, we were able to get 15 realizations which provided a kinetic energy spectrum which was similar to the one we have put in the manuscript but with more fluctuations. Currently we have doubled the number of realizations which provides us with better results.

- We agree with the referee that the energy spectrum of a turbulent active nematic is very well known in the literature. We have now presented this as a supplementary information.

.11. Vertical and horizontal span in the definition of parameter A(t) measuring droplet deformations sensibly depend on the reference system. Why not using a parameter measuring the sphericity of the droplet, independently of the reference system?

- We thank the referee for the suggestion. We agree that A(t) is one of several choices. From the interface location data available from our front tracking algorithm, we were able to accurately obtain the spans of the droplet. Further, in our simulations with the different droplet sizes, the surface tension was in a range where the deformations were not massive. Therefore, for the purpose of our simulations and the description of our results, A(t) proved to be a good measure.

12. The authors claim that the spectral plots in Fig. 2d exhibit nearly 1⁄ω decay. I am not convinced. Can the authors provide fit with errors? Analogous considerations apply for other scaling laws reported in Fig. 2e,f.

- The lines showing the decay of the spectral plots are used as a guide to the eye in the manuscript. We do agree that in the earlier version we did not have enough averaged data and for multiple particle sizes to clearly show the behavior. We now have data for more radii and have averages over more realizations. We hope that the reviewer is convinced now about the $1/\omega$ and $1/\omega^3$ scaling in the revised Fig. 2.

13. How are the spectra S(ω) defined? On a related notice, the quantities ω_Δr,ω_Δθ,ω_A in Fig. 2d,e,f are also undefined. I believe the authors mean that ω is the Fourier frequency, which should not be labelled, while subscripts should be used to label the corresponding spectra S_Δr,S_Δθ,S_A.

- $S(\omega)$ is defined as the power spectrum corresponding to the various time series. We did indeed mean that ω is the Fourier frequency. We agree with the reviewer that we should not have labelled them differently and rather labelled the spectrum. In the revised manuscript, we have made the corrections as suggested.

14. At line 150, what is d(r)? I could not find its definition anywhere.

- If we understand the remark correctly then the quantity the referee is mentioning is the differential $d(\Delta r)$ where Δr is the variable of integration.

15. In Fig. 2a-c on the x-axis is t the dimensional or dimensionless time? It would be advisable to compare the time normalized by the active timescale ζ⁄η. Analogously the y-axis in Fig. 1a could be normalized with respect to the active lengthscale √(K⁄ζ).

- In the revised manuscript, we now clearly mention the length and time scales in the caption of Fig.1 as well as in the Methods section. The scales were mentioned in the “rescaling of the governing equations” section before but we agree that they must be mentioned in the text and figure captions for clarity.

16. The author claim that Fig. 3a shows the existence of a diffusive regime, but I cannot see it from the figure. Could the author provide evidence?

- We have now performed several additional simulations to address this issue. For all simulations, we now have increased the system size to address boundary effects and also reached simulation times > 1000. For all sets of data, we now look at the transition to a diffusive regime. For particles with larger radius, where the diffusive regime sets in earlier, we clearly see a very good fit to a diffusive scale. We do regret however that even after the improvement of the data, we still cannot provide the exact value of the exponent of the MSD power law for all different values of the radius and surface tension after the ballistic regime and therefore state that it is $1 < \delta < 2$. It is however clear now that there is a transition from the ballistic regime. In Fig. 3(a) the transition time depends on R but in the new Fig. 4(i) it turns out that the transition time does not depend so much on the surface tension. Note that now in the improved plots we have excluded data for$t < 300$ i.e. t_0 is set to 300 in both Fig. 3(a) and Fig. 4(i).

17. I am also concerned about the fact that the authors considered only 3 values for the droplet radius. I believe that more values should be considered in order to draw significant conclusions.

- We agree with the referee on this point. To address this issue, we have now done our study for 8 values of droplet radius and for different values of surface tension. The number of realizations that we have used is also double that we had earlier. Although this involved a large number of very expensive runs, we are happy to state that we have now a much larger set of data which corroborates our conclusions. We have discussed the same in the manuscript. We hope that this addresses the concern of the referee with regards to our conclusions.

18. In the conclusions the authors claim that the transition from the superdiffusive to the diffusive regime also depend on the properties of the active bath. However, from what what reported in the manuscript and in Table I the authors only varied the droplet's radius. Indeed, it would be interesting to test the behavior of the system at varying the activity considering both extensile and contractile activity. This is a point that an article on the anomalous dynamics of a passive droplet in active turbulence should not neglect.

- We agree with the referee that the dependence on the activity should be probed carefully to see the dependence of the transition from the superdiffusive to diffusive regime on the properties of the active bath. Our main motivation for the study was to see the dynamics of a deformable droplet in an active turbulent fluid and characterize its dynamics. Therefore, we focussed our study to see how the variations of the droplet size and surface tension affects the dynamics in the active medium. We believe that for that purpose we have provided a significant number of interesting results verifiable under experimental conditions. Therefore for the current study we could limit ourselves to an extensile active nematic system for an activity where turbulence is observed. Our statement regarding the transition depending on the properties of the active bath was based on our MSD results and the experimental observations of dynamics of colloids in bacterial baths. We have accordingly revised the

manuscript. We believe that we have an excellent test bed for future study where all such behavior should be probed in greater detail.

Reviewer #2 (Remarks to the Author):

The dynamics of a passive droplet suspended in active nematics subjected to friction is studied in this manuscript and the effect of droplet size is analysed. It is observed that the droplet moves almost ballistically for longer time scales and that it moves faster for smaller radii. The problem is interesting and could help explain phenomena in biological systems such as the motion of tracers and other particles in bacterial swarms. However, in the opinion of this referee, this work should be submitted to a more specialised journal after some revision and verification of the results (suggestions below). Many previous works have investigated the dynamics of droplets in active nematics with some of them being acknowledged in the introduction. For instance, in [Soft Matter, 10, 7826-7837(2014)] it was found that the passive droplets might acquire directed motion due to the formation of topological defects close to the interface (do you observe them in your simulations?). Other works have studied the dynamics of active droplets in a passive fluid [e.g.: Nat. Phys. 17, 260–266 (2021), Phys. Rev. Research 5, 033165 (2023), Soft Matter, 13, 5933-5941 (2017)]. Thus, the present study is original to the best of my knowledge but is an extension of previous works in the literature.

- We thank the referee for the critical review and the observation that the work is interesting and that it could explain phenomena in biological systems. In the following, we provide a detailed rebuttal.

- We first thank the referee for the reference of G. Magistris et. al. Soft Matter (2014). While we had tried to provide an exhaustive reference list of previous work, we had missed this very relevant reference. We however respectfully disagree about the comment that there have been many work which has investigated the dynamics of droplets in active nematics. The inverted problem (active droplets in passive fluid) has a completely different perspective to ours and therefore although we have cited them in our manuscript, we refrain from discussing them here. There are only two papers of significant relevance in the context of our work in the last decade which have explored a passive nematic droplet in an active fluid.

- The first by Foffana et. al. (Phys. Rev. Lett. (2012) which we had referred in our manuscript) studied a very interesting question about what happens when a passive particle of radius R is dragged through an active nematic with a force F. This question, relevant in the context of rheological experiments, showed that the drag coefficient $\gamma = F/v$ (with v a steady particle speed) exhibits a strongly nonlinear dependence on radius, in violation of Stokes' law. This phenomena was explained in terms of the deformation of the orientational order in the active fluid around the probe particle. Another crucial observation was that in a contractile fluid the colloidal probe should move steadily in the direction opposite to the applied force, i.e. a stable steady state with a negative drag coefficient. The spherical colloidal probe was modelled by a standard method of bounce-back on links which provides a no-slip boundary condition for the velocity field at the solid surface. While the surrounding fluid was active it was naturally not in the turbulent regime as the context of the problem was completely different.

- The second work by Magistris et. al. Soft Matter (2014) (which the referee has kindly pointed out), is closer to our work as the dynamics of a passive droplet was observed in the absence of a pulling force. In this work however, the passive droplet considered was an isotropic one and the active fluid was not in the turbulent regime. Their study showed how the variation of activity and anchoring conditions (both normal and tangential) of the polarisation of the active gel at the droplet surface leads to different dynamics of the droplet. While this study is interesting in its own right, the general premise of this study is very different from our work. Please note that the follow up work by Tiribochhi et. al. Soft Matter (2016) was discussed in the manuscript.

- The main motivations of our work instead was to study vesicle deformation fluctuations and dynamics in an active cytosolic environment. It is to this effect that we looked at a droplet whose surface tension can be varied. Recent experiments of rigid mobile inclusions in an active fluid (formed using microtubule based active nematics) showed that the chaotic motion of the fluid can be harnessed to rotate milli-meter sized propellers and pinwheels (Ray et. al. PRL (2023), https:// physics.aps.org/articles/v16/97). The intracellular particles in biological active fluids are on the other hand deformable in nature. How does one understand the interactions between active fluids and movable and deformable boundaries? We boldly claim that this is the first such study of a deformable particle in a turbulent active fluid. Our study apart from being novel and biologically relevant, has the following distinct features: (1) We are able to make the best possible connection with experimental studies as our study explicitly incorporates hydrodynamics of the fluid and the droplet. We reproduce the short time super-diffusive dynamics of the droplet as seen in reported experiments. (2) Theoretical understanding of how to design colloidal systems to produce a desired dynamical response is lacking, and our study demonstrates for the first time how an inclusion with fluctuating shape moves in an active turbulent environment. (3) Theoretical studies mostly rely on a Langevin framework which ignores hydrodynamics. We have well resolved solutions of the velocity field and therefore can characterise the spatial and temporal correlations and associated length and time scales in the active bath which affect the dynamics and deformations of the droplet. (4) We are able to study the influence of the active bath when the size of the droplet is changed across this length scale – particularly, a shift from normal diffusive trajectories to periods of persistent motion and stall of the droplet. (5) We have looked at the variation of the surface tension of the droplet and show that at very low values of the surface tension the droplet begins to break and mix with the outer active bath.

- To the best of our knowledge, it has not been explored before how the motion of passive inclusions in active turbulence is influenced by the interplay of spatial correlations of the flow and the size of the inclusions. We expect that this study might inspire experiments in the research field and further theoretical investigations.

- We earnestly hope that the reviewer would reevaluate their position and give the paper serious consideration for publication in this journal in light of the previously stated arguments and the thorough modification that we have made.

Regarding the results, the MSD in Fig. 3b should be double-checked. This ballistic behaviour could be an indication of finite-size effects. Because you simulated a relatively small system

(200X200 or 400X400) the active nematics could be forming directed flows due to the periodic boundary conditions instead of random vortices. The authors should check if this ballistic regime persists for different system sizes (or frictions) and with a closed system instead of periodic ones.

- We thank the reviewer for pointing this out.

- To support our results, we have now performed two additional set of simulations – (1) increasing the set of droplet radius R, and (2) varying the interfacial tension – a total of 2x140=280 simulations. Also in all the new simulations we increased the system size to 450x450 with grid size 576x576 keeping the overall simulation time manageable. This we believe has helped to mitigate, or at least reduce, the boundary effects on the droplet trajectories once they go closer to the boundaries, to get better MSD and VACF data. Also, we have managed to reach simulation time > 1000 for each new simulation and now the transition from the ballistic to diffusive or superdiffusive regime is quite clear in Fig. 3(a) with varying R, and in Fig. 4 with varying surface tension. We cannot provide the exact value of the exponent of the MSD power law after the ballistic regime and therefore state that it is $1 < \delta < 2$. It is however clear now that there is a transition from the ballistic regime. In Fig. 3(a) the transition time depends on R but in Fig. 4 it turns out that the transition time does not depend so much on surface tension.

- The vorticity and velocity fields shown in Fig. 1 in the developed state clearly show sufficient number of vortices and minimizes the possibility that active nematics could be forming directed flows due to the periodic boundary conditions instead of random vortices.

It is not completely clear that the energy spectrum in fig. 1f follows a -4 power law at the end. Anyway, the authors should also check if the -1 and +1 power laws for small and intermediate wave numbers are found. Depending on the friction, other power laws can be observed [Soft Matter, 16, 4256-4266 (2020)].

- We believe that the energy spectrum obtained in the relevant Reynold's number regime clearly shows a -4 power law scaling. The slight deviations at larger k values are fluctuations due to limited averaging. Increasing the averaging over initial conditions reduces these fluctuations. We agree with the reviewer that depending on the friction, other power laws can be observed. However, these simulations were run to benchmark our code and we put these plots to show reproducibility of our results. These plots are now put in the supplementary.

What is the method used to solve the equations for the dynamics (Navier-Stokes and Beris-Edwards)?

- We have now revised the description in the methods section so that the methods and algorithms used to solve the Beris-Edwards dynamics with droplet is clear to the reader. Please refer to Methods section and sub-sections therein in the revised manuscript.

It is said that the droplet follows "stays" and "runs". Is it the same as the Levy walks observed in [Phys. Rev. Lett. 127, 118001]?

- We thank the reviewer for this interesting remark. We are not sure if the “runs” and “stays” observed in our system are same as the ones observed in typical Levy walks. To avoid any speculation, in the manuscript we avoided discussing this point. Although it would be interesting to explore, we save it for our future work, as pursuing this line of thought would have altered our broader objectives of the study i.e. what is the effect of droplet size and deformability on the dynamics of the droplet trajectories in an active bath.

Reviewer #3 (Remarks to the Author):

This theoretical study investigates the dynamics of a passive deformable droplet in active nematic turbulence described by the standard partial differential equations for active nematics. The droplet exhibits fluctuating deformations and movement. Here, the mean-square displacement shows a superdiffusive regime that transitions to diffusive behavior for large time scales. The main result of the study is that the radius is very important in this context. When it is smaller than the correlation length of the velocity field, the droplet experiences strong advection, whereas when it is larger, the droplet will remain more localized. The authors end the manuscript with an effective Langevin description of the droplet motion that qualitatively captures the temporal correlations observed.

Overall, the manuscript is well written and the data is presented in great detail. The main result concerning the droplet size compared to spatial correlations of the surrounding flow field is indeed very interesting. To the best of my knowledge, it has not been explored before how the motion of passive inclusions in active turbulence is influenced by the interplay of spatial correlations of the flow and the size of the inclusions. I expect that this study might inspire experiments in the research field and further theoretical investigations.
However, there are a few key points that in my opinion should be addressed before publication. These concern the actual role of the deformability of the droplet, the correspondence to the effective Langevin description and overall the limited data set varying only one parameter. Therefore, I would recommend publication in a more specialized journal.

- We would like to thank the reviewer for the positive comments about the manuscript and considering it for publication. We are also happy with the assertion that this study might inspire experiments in the research field and further theoretical investigations, which was also the main motivation of the work. To convincingly address the concern of the reviewer regarding the limited data set varying only one parameter and for a few values of particle size, we are happy to state that we have now performed a series of extensive simulations. In the revised manuscript, we now present results for (i) 5 additional sets of radius of the droplet and (ii) for a set of surface tension values. In addition, we have increased the system size to 450x450 with grid size 576x576 while keeping the overall simulation time manageable. We believe that this has helped to mitigate, or at least reduce the boundary effects on the droplet trajectories and to get better MSD and VACF data. Further, we have managed to reach simulation time > 10^3 for each parameter variation. We are happy to note that we have been able to address two major criticisms of the reviewer - the actual role of deformability of the droplet and that of a limited data set - in great detail. With this extensive set of data and the resulting analysis, we sincerely hope that the reviewer finds the current version of the manuscript suitable for publication in this journal.

- Below we provide a point by point response to the reviewer comments.

In the following, I explain my main concerns and comments that should be addressed before publication.

Major comments

1. The authors stress that the deformability of the droplet is taken into account. However, they never really address the impact of deformability on their results. They mostly show how the size of the droplet changes its motion, which is a very interesting result in its own right. But I wonder whether the same effect would be observed when looking at a rigid inclusion. What happens if the deformability (interfacial forces) is varied?

- We thank the reviewer for this valuable comment. In the revised manuscript, we have now added results where the interface tension is varied over six different parameter values. We observed that the variation of Re_I does not change the VACF and MSD significantly. However, when Re_I is significantly reduced, we observe that the droplet begins to break and mix with the active bath. We would also like to mention that we have now eight different values of the droplet radius. The ensemble averaged total length of the trajectory in a given period comes out to be inversely proportional to the radius of the droplet, which endorses our claim that smaller droplets travel longer distances. Please refer to subsection "Effect of interfacial tension" in the revised manuscript, the new Fig. 4 and the supplementary plots.

2. The authors often talk about the transition from the superdiffusive to the diffusive regime without ever really showing the transition in the MSD in the figures. At least, a line showing diffusive behavior ~(t-t_0) should be added to Fig. 3 (b). If the transition is not really visible in a clear way, it might be advisable to increase the length of the simulations and the number of times the simulations are repeated (15 times seems hardly enough to calculate an MSD). If this is not feasible for computational reasons, the authors should justify such a low number of repetitions.

- We thank the referee for pointing this out. We have now doubled the number of times the simulations are averaged and also reach simulation time > 1000 for each new simulation. The transition from the ballistic to diffusive or superdiffusive regime is much better observed in new Fig. 3(a) with varying R, and additionally in Fig. 4(i) with varying Re_I. We would like to point out that for the largest radius $R = 64$, we are able to see the transition to the diffusive regime clearly. For the smaller radius, although we clearly see a shift from the ballistic regime, we cannot certainly state that the diffusive regime is reached. Rather, for such situations, $1 < \delta < 2$. With our current computational resources, we have not been able to run for longer times to clearly observe the transition for all the droplet sizes. In the variation of Re_I for a fixed radius, the transition to the diffusive regime is much clearer (see new Fig. 4).

- We would like to comment that while the transition between the ballistic and diffusive regime for the isotropic Brownian motion of a colloidal particle in a liquid bath is exponential, a rigorous hydrodynamic theory (E. J. Hinch, J. Fluid Mech. 72, 499 (1975), Weitz et. al. Phys. Rev. Lett. 63, 1747 (1989)) predicts that the approach to the asymptotic diffusive regime is much slower which is observed in our simulations. This is also reflected in the velocity auto-correlation (VACF) which shows long time tails (see Fig. 3(f)).

3. Concerning the effective Langevin description, the authors should state where their approach differs from earlier approaches to describe passive tracers in active baths or if it is indeed equivalent to these earlier approaches, in particular concerning the results derived in the Methods section. Tracers in active baths have been modeled before via generalized Langevin equations with instantaneous friction and noise with memory kernel, see for example Wu, Libchaber, Phys. Rev. Lett. 84, 3017 (2000) or Maggi, Paoluzzi, Pellicciotta, Lepore, Angelani, Di Leonardo, Phys. Rev. Lett. 113, 238303 (2014). Further, the dynamics of active particles themselves have been modeled in a similar way, see for example Bechinger, Di Leonardo, Löwen, Reichhardt, Volpe, Volpe, Rev. Mod. Phys. 88, 045006 (2016). The memory is sometimes assumed to be generated by an Ornstein-Uhlenbeck process [G. E. Uhlenbeck, L. S. Ornstein, Phys. Rev. 36, 823 (1930)]. Is the approach used by the authors equivalent to these earlier approaches? If yes, this point should be mentioned and references should be given.

- We thank the referee for this insightful comment and we agree that the point is somewhat unclear in the manuscript. Our approach is indeed equivalent to for example the work by Wu and Libchaber with instantaneous friction and noise with memory kernel to describe the bath. As the referee mentions correctly, all the papers also use similar approaches, while concluding the difference from passive Brownian motion. Therefore, we have now completely rewritten this section of the manuscript to make clear connection with the existing results (see the section Effective Langevin description and the Methods section). In our study, we are able to show that if we study the outer bath in a resolved manner such as by solving the Beris-Edwards equations for the bath, we can extract certain spatial features or scales, and when compared to the size of the inclusion, these scales can dictate the dynamics of the inclusion – for instance our result that smaller inclusions travel longer distances. We now make these note explicit in the manuscript and refer the papers at the end of the section “Effective Langevin dynamics”.

4. To my understanding, the effective Langevin description can only qualitatively reproduce some aspects of the droplet motion, however, it has no predictive power concerning the effect of the droplet radius as the radius might influence many of the coefficients in the effective Langevin description. Thus, in the current form the Langevin model does not explain the most interesting aspect of the observations. Here, it should be stated more clearly what the benefit is of introducing the effective description at all. To increase the significance of the effective description, might it be possible to map the dynamics obtained from the numerical calculations to the effective Langevin model? For example, can an effective temperature be calculated? In my opinion, this would be an interesting point because effective temperatures have been calculated for active systems before and usually increase with increasing activity. What is expected in the present system? How does the radius of the droplet impact the effective temperature?

- We completely agree with the referee that in the current description, the effective Langevin description can only qualitatively reproduce some aspects of the droplet dynamics. We are currently in the process of building a hydrodynamic theory to understand the dynamics of the droplet. While we have some preliminary results which are encouraging, we do not have a complete picture to report as of now. The determination of an effective temperature from the hydrodynamic simulations has proven difficult and therefore within the effective Langevin description, we have not pursued this approach. In the revised manuscript, we have therefore removed the discussion on an effective temperature. From the theoretical analysis of the effective Langevin description with a memory which decays exponentially in time (see Methods section and the Effective Langevin description),

$$\langle F(t')F(t'')\rangle = \Lambda \exp[-|t'-t''|/\tau_F], \langle F(t)\rangle = 0.$$

one can obtain a definition of an effective temperature from the active component of the diffusion coefficient which is given as $D_a = \Lambda\tau_F/\alpha^2$, where α is the coefficient of friction. The effective temperature is therefore $k_B T_{eff} = D_a\alpha = \Lambda\tau_F/\alpha$. Unfortunately, although the fluctuation-dissipation (FDT) relation-based effective temperature can be defined for the active bath, it is not clear what properties of the active bath it actually signifies.

5. Why is the absolute step size used when calculating the step autocorrelation function and not the step vector? Step sizes with the same magnitude but opposite direction are thus perfectly correlated. I understand that the angular steps are also defined and cover this information. However, the correlations of these angular steps are never determined. My main concern here comes from the fact that the step autocorrelation time is later compared to the velocity autocorrelation time obtained via the effective Langevin dynamics, which is not calculated from the absolute velocity but from the velocity vector.

- We sincerely appreciate this observation by the referee. We have now corrected our definition, as well as calculations, to include the step vector instead of the step size while computing the VACF. Indeed, this correction has helped us distinguish multiple time scales associated with VACF which change when we change R but not so much when we change Re_I for the surface tension. Please refer to new plots on VACF in the revised manuscript in Fig. 3 and Fig. 4.

6. The authors should cite theoretical investigations of tracer particles in active fluids such as bacterial suspensions described by continuum-theoretical models. In particular, there are a few works that investigate the impact of flow patterns in active turbulence on tracer dynamics: Sanjay, Joy, Phys. Rev. Fluids 5, 024302 (2020), Mukherjee, Singh, James, Ray, Phys. Rev. Lett. 127, 118001 (2021), Reinken, Klapp, Wilczek, Phys. Rev. Fluids 7, 084501 (2022). These should be cited, e.g., in l. 38. Further, there are theoretical attempts to justify the superdiffusive regime observed in bacterial turbulence, which should be cited in ll. 154F: Ariel, Be'er, Reynolds, Phys. Rev. Lett. 118, 228102 (2017).

- We would like to thank the reviewer for pointing out important literature on the subject which we had inadvertently missed. We have now cited the above mentioned relevant

papers in appropriate context in the revised manuscript.

Minor comments:

1. The authors use an underdamped Langevin equation for the effective description. I wonder if an overdamped description would not be sufficient in this case. Is the inertia of the droplet really important here? Taking an overdamped description would significantly simplify the calculations in the Methods section. The reduced number of independent parameters might also help to find a better correspondence between the observations from the numerical simulations and the effective Langevin description.

- It is indeed true that we could have used the overdamped description as we are looking at low Reynolds' number turbulence. However, we opted to use the underdamped equation for generality and to look at role of different time scales in the problem. We note that the velocity auto-correlation function can now be directly compared as per the suggestions of the referee. We are also pleased to note that we have shown one comparison where the velocity of the droplet decreases linearly with the radius (Fig. S3 in the supplementary) which we can extract from our theoretical study.

2. The introduction of the initial state is somewhat confusing (l. 91). Is the initial state isotropic or nematic?

- In our simulations, the initial state is a director field oriented along a particular direction (nematic) and with a random perturbation. This is now clearly stated in the manuscript.

3. A more detailed introduction to how the interfacial forces are calculated in Eq.(4) is needed to really understand the model. This should be given in the Methods section.

- We completely agree with the suggestion and apologize that this was not clarified in detail in the manuscript. We have added a detailed description of calculation of interfacial force in the revised manuscript.

4. What is the significance of Fig. 3 (a)? Can the behavior in the MSD for smaller values of time lag only be observed because the point t_0 here is chosen as the beginning of the simulation when turbulence has not developed yet? If this is the case, I would not show this -plot and only show (b) where t_0 is chosen in the fully developed turbulent state.

- We are sorry for this confusion. We have now corrected this as suggested by the reviewer in Fig. 3(a).

5. I think it is not necessary to show Fig. 3 (f). This could be shifted to a Supplemental Material or not shown at all. Not showing Fig. (a) and (f) would also allow to increase the size of the other figures, which are too small in my opinion.

- - We agree with the reviewer regarding Fig. 3(a). We have now removed this plot. In the revised Fig. 3(a) we have the MSD for a long range with the more extensive simulation runs. We have however kept the variation of the integral length scale with time plot. We also have new plots of the VACF as suggested by the referee. Together, we hope that the revised plot is both informative and clear.

6. Similarly, I would suggest to split Fig. 1 up into two figures to increase the size and readability.

- We thank the referee for the suggestion. We have now removed the plots of the kinetic energy spectrum and put them in the supplementary as they were essentially benchmarking the turbulent state. With this and further changes in the arrangement, we hope that Fig. 1 is readable.

7. It should be better justified why \tau_{\Delta r} obtained from the step autocorrelation function in the simulation corresponds to the correlation time obtained via the velocity correlation function in the effective Langevin description (see ll. 186ff). After all, one quantity characterizes correlations in the step size, the other velocity correlations.

- We have now corrected the calculations of VACF using the step vector as suggested by the reviewer (see earlier comment), and have made the necessary changes.

8. I think it would benefit the manuscript if the fluctuation-dissipation theorem and equilibrium versus non-equilibrium is discussed in the context of the effective Langevin description. My understanding is that the FDT is not assumed to hold from the outset (Eqs. (14) and (15)), which is indeed not a necessity as we are dealing with a non-equilibrium system. However, an effective temperature is introduced between Eqs.(20) and (21). Does this introduce an effective equilibrium assumption? I suggest discussing this point in the main text around l. 186 and in the Methods section around l. 310.

- We have now discussed this in reply to an earlier comment by the reviewer (see Major comments: Answer to comment 4).

9. The introduction of the model equations and the rescaling procedure is shifted to the Methods section, which helps in keeping the manuscript accessible. However, in l. 110, when the explored range for the radii of the droplet is given, there has to be some kind of scale given as well. What does a radius of 15 mean? Relative to what? At least in the case of the radius the scaling has to be discussed briefly in the main text as well.

- The scales are now explicitly mentioned wherever necessary in the revised manuscript.

10. The sentence in ll. 142ff is confusing. Is it supposed to mean "... have dynamics distinct from the motion increments."?

- We have now corrected the statement.

REVIEWER COMMENTS

Reviewer #1 (Remarks to the Author):

In the amended version of the manuscript, the authors have fully addressed the concerns of both referees regarding some model features and the robustness of the simulation results. In particular, the authors have made considerable efforts in exploring different model parameters, varying surface tension and droplet radius, and providing solid numerical evidence supporting the claim of a transition from ballistic dynamics to normal diffusion depending on the droplet size. Furthermore, the overall quality of the model presentation and result discussion has considerably improved. As I already mentioned in my initial review, I believe that the results presented here will be of interest to the community working on active turbulence and active matter in general. For these reasons, I endorse the publication of the article in Nat. Commun. in its present form.

Reviewer #2 (Remarks to the Author):

In the revised manuscript, the authors have addressed most of my concerns and have improved the manuscript. They claim that this is the first study investigating the motion of flexible particles (droplets in this case) in active turbulence, which I agree to the best of my knowledge. The previous studies I am aware of treated either tracers in active turbulence or droplets in non-turbulent flows.

Other questions:

It is mentioned throughout the manuscript (in the first paragraph of the Discussion section for instance) that the interaction between inclusions and active fluid is studied. However inclusions are usually solid and involve no-slip conditions at the fluid-solid surface (e.g., https://arxiv.org/abs/2307.11587 and Ref. 42). In this manuscript, the authors study droplets, which have different boundary conditions (continuous velocity and shear). Do the authors think the conclusions will be valid for solid suspended bodies?

In Fig. 3a, the regime apparently becomes diffusive for R=8 and 64 (the extremes), but, strangely, intermediate values do not have this clear transition (16, 32 and 48). What is the explanation?

For which conditions does the droplet break? Can you give the corresponding active capillary number?

Reviewer #3 (Remarks to the Author):

I would like to thank the authors for their detailed answers to the points I raised regarding the manuscript. All of my concerns have been addressed and the changes to the manuscript are all adequate. The authors significantly extended their study and performed additional numerical calculations to increase the statistics and include a variation of the droplet deformability. These new results complement the manuscript very well.

With these extensions, I think the manuscript is suited for publication in Nature Communications. However, to obtain my full support, the following minor points should be addressed:

1. I like the explicit introduction of the active stress in ll. 100. I think it it would be further beneficial to quickly discuss the sign of the coefficient at this point and point out that the study is concerned with extensile active nematics (in contrast to contractile) already here in the main text.

2. l. 111: Weird notation of the scalar product. Dot is at the bottom of the line. I suggest consistent notation (as in l. 140).

3. What is the value of the time step? As the quantities shown in Fig. 1 are determined as changes in one time step, I think the value of the time step should be given either in the caption of Fig. 1 or in the main text (l. 139).

4. The meaning of \tau_m should be already introduced in main section (l. 218).

5. Typo in l. 222: average

6. Undefined reference to equation in the caption of Fig. 3.

RESPONSE TO REVIEW REPORT: MANUSCRIPT NCOMMS-23-41742A

Summary of main revisions in the manuscript:

We thank all the reviewers for their comments. We have made all the minor changes as per the comments of the reviewers. These changes include

(i) A few additional lines to answer some of the comments of the reviewers and minor typos which were kindly pointed out by the reviewers.

(ii) Some labels were missing in the earlier plots. These have now been provided in the current manuscript.

(iii) Figure 3(b) inset is now changed.

Below we provide point by point response to the referee comments.

Reviewer #1 (Remarks to the Author):

In the amended version of the manuscript, the authors have fully addressed the concerns of both referees regarding some model features and the robustness of the simulation results. In particular, the authors have made considerable efforts in exploring different model parameters, varying surface tension and droplet radius, and providing solid numerical evidence supporting the claim of a transition from ballistic dynamics to normal diffusion depending on the droplet size. Furthermore, the overall quality of the model presentation and result discussion has considerably improved. As I already mentioned in my initial review, I believe that the results presented here will be of interest to the community working on active turbulence and active matter in general. For these reasons, I endorse the publication of the article in Nat. Commun. in its present form.

- We are extremely happy that the reviewer has endorsed the publication of the article in its present form. We would like to thank the reviewer for the valuable criticisms and comments that have helped to signficantly improve our manuscript.

Reviewer #2 (Remarks to the Author):

In the revised manuscript, the authors have addressed most of my concerns and have improved the manuscript. They claim that this is the first study investigating the motion of flexible particles (droplets in this case) in active turbulence, which I agree to the best of my knowledge. The previous studies I am aware of treated either tracers in active turbulence or droplets in non-turbulent flows.

- We are glad that the reviewer has agreed to our assertion that this is the first study investigating the motion of droplets in active turbulence and the positive response to our manuscript.

Other questions:

It is mentioned throughout the manuscript (in the first paragraph of the Discussion section for instance) that the interaction between inclusions and active fluid is studied. However inclusions are usually solid and involve no-slip conditions at the fluid-solid surface (e.g., https://arxiv.org/abs/2307.11587 and Ref. 42). In this manuscript, the authors study droplets, which have different boundary conditions (continuous velocity and shear). Do the authors think the conclusions will be valid for solid suspended bodies?

- We thank the referee for this interesting point and we agree that the velocity and shear boundary conditions on the surface of a solid inclusion will be different than the present case of a deformable droplet. The droplet adjusts its shape in response to the forcing by the external bath, and thus simulating the dynamics would be different than a solid inclusion. However in the limit of increasing Re_I, we believe that the dynamics effectively simulates, atleast qualitatively, a solid inclusion. Having said that, we however leave this interesting question asked by the referee for a possible future exploration (see below). We thank the referee and briefly mention this point in the methods section of the revised manuscript.

- The application of the method that we have used to model the droplet poses problems when considering applications to flows with rigid bodies as the constitutive laws are not generally well posed in the rigid limit. As mentioned above, one way to circumvent the problem would be to look at the large Re_I limit. Another method developed by Goldstein et. al. J. Comp. Phys. (1993), simulates the effect of the rigid body on the surrounding flow via a forcing term $\vec{F}(t) = \alpha \int_0^t \vec{u}(\tau) d\tau + \beta \vec{u}(t)$, where the coefficients α and β are chosen to enforce the boundary conditions. This method was used to simulate flow past solid objects at moderate Reynolds number and low Reynolds number turbulent flows.

In Fig. 3a, the regime apparently becomes diffusive for R=8 and 64 (the extremes), but, strangely, intermediate values do not have this clear transition (16, 32 and 48). What is the explanation?

- We thank the reviewer for this important question which allowed us to look back at the data carefully. The diffusive regime sets in faster for the larger particle which is expected. For the smallest particle size considered, $R = 8$, the apparent transition to a diffusive regime is incidental. At this particle size, for some of the realizations, the particle reached the boundaries at long times giving rise to boundary effects. For these large time scales, the data for the MSD was therefore averaged only over those realizations where the particle was well inside the box. We could have used the same number of realizations for the entire time of the MSD, however, the mean for the short times gives much better statistics with the higher number of realizations. This issue can be circumvented by choosing much bigger box sizes which however is computationally expensive. We do however expect the crossover times to correlate with the size as was shown for solid passive tracers in active bath [Wu et. al. PRL 2000].

For which conditions does the droplet break? Can you give the corresponding active capillary number?

- We thank the referee for this comment. As we have shown in Supplementary Fig.4, the transition happens around $Re_I = 1$. Indeed upto around $Re_I = 3$, we did not observe a mixing. The capillary number is defined as the inverse of Re_I in the dimensionless units we have chosen. Please note that this breakup has been investigated for a given value of activity and droplet radius. Generic conditions for the breaking up of the droplet is an interesting question in itself and could be part of future work.

Reviewer #3 (Remarks to the Author):

I would like to thank the authors for their detailed answers to the points I raised regarding the manuscript. All of my concerns have been addressed and the changes to the manuscript are all adequate. The authors significantly extended their study and performed additional numerical calculations to increase the statistics and include a variation of the droplet deformability. These new results complement the manuscript very well. With these extensions, I think the manuscript is suited for publication in Nature Communications. However, to obtain my full support, the following minor points should be addressed:

- We would like to thank the reviewer for the constant support, time and constructive feedback which has helped us to improve our manuscript. We have made the necessary minor changes that the reviewer has raised.

I like the explicit introduction of the active stress in ll. 100. I think it it would be further beneficial to quickly discuss the sign of the coefficient at this point and point out that the study is concerned with extensile active nematics (in contrast to contractile) already here in the main text.

- We thank the reviewer for the suggestion. We agree with the reviewer and have now discussed that our system is extensile, right after introducting the active stress, in line 103 in the revised manuscript.

2. l. 111: Weird notation of the scalar product. Dot is at the bottom of the line. I suggest consistent notation (as in l. 140).

- We have corrected the notation as per the suggestion of the reviewer.

3. What is the value of the time step? As the quantities shown in Fig. 1 are determined as changes in one time step, I think the value of the time step should be given either in the caption of Fig. 1 or in the main text (l. 139).

- We thank the reviewer for pointing this out. We have now provided the value of the time step in the caption of Fig.1 as suggested.

4. The meaning of \tau_m should be already introduced in main section (l. 218).

- We have now introduced the meaning of τ_m earlier in the main section in line 221 in the revised manuscript.

5. Typo in l. 222: average

- We have fixed this typo in the revised manuscript.

6. Undefined reference to equation in the caption of Fig. 3.

- We thank the referee for bringing this up. We had overlooked the correction of this inset in the light of our calculations when we edited the last version of the manuscript. We have

now updated the manuscript with the inset which shows the variation of the MSD as a function of t/τ_F for various values of τ_m mostly in the limit of $\tau_m \ll \tau_F$. The approximate MSD in this limit is also given in the methods section and the section is referred..

REVIEWERS' COMMENTS

Reviewer #2 (Remarks to the Author):

The authors have satisfactorily addressed my questions and concerns.
